# Detection of adulterated drugs in traditional Chinese medicine and dietary supplements using hydrogen as a carrier gas

**DOI:** 10.1371/journal.pone.0205371

**Published:** 2018-10-10

**Authors:** Yen-Ping Lin, Ying-Lin Lee, Chien-Ya Hung, Chuan-Fa Chang, Yi Chen

**Affiliations:** 1 Public Health Bureau, Tainan City Government, Tainan, Taiwan, R.O.C; 2 Department of Medical Laboratory Science and Biotechnology, College of Medicine, National Cheng Kung University, Tainan, Taiwan; Indian Institute of Chemical Technology, INDIA

## Abstract

Helium, a minor component of natural gas and radioactive minerals, is most commonly used as a carrier in gas chromatography-mass spectrometry (GC-MS). Its scarcity leads to limited availability and higher costs. In this experiment, hydrogen from a safe source of a hydrogen generator was tested as a substitutive carrier gas for the detection of adulterant in traditional Chinese medicine (TCM) and food supplements by GC-MS analysis. We found that the limits of detection (LODs) of using hydrogen were from 10 to 1000 μg/g. The levels of LODs tested among 170 drugs remain the same whether hydrogen or helium was used as a carrier gas with the exception of 7 drugs—benzbromarone, estradiol benzoate, bezafibrate, mefenamic acid, oxymetholone, piperidenafil and cetilistat. The real sample analysis results using hydrogen were as satisfactory as those using helium. In addition, the retention time was shortened after the chromatographic performance was optimized. In summary, it is worth considering hydrogen as a carrier gas due to its affordable costs, energy efficiency, carbon reduction and chromatographic advantages to detect adulterated drugs in TCM and dietary supplement using GC-MS.

## Introduction

Currently, TCM and dietary supplements are becoming progressively popular in many countries [1]. The worldwide consumption of TCM and dietary supplements has accumulatively increased. Evidence showed that approximately 80% of the world’s population takes herbal medicines. In general, TCM is generally regarded as possessing few side effects, and users are therefore prone to taking it in excess of the recommended dose for an extended period of time. On the other hand, consumers tend to trust natural products, believing that they are safe and free of side effects. This false belief that TCM and food supplements are all natural with no side effects and that they are not harmful to human health has led to the exponential growth of TCM and food supplement remedies in markets worldwide [2–4].

To enhance their efficacy, TCM and food supplements were often illegally adulterated with western medicine. The quantities of adulterants in TCM and food supplements at times even exceeded the normal dosage range, and in many cases, they were not the ones required or responsible for the therapeutic effects advertised on the label [3, 5, 6]. Recently, various scientific and monitoring investigations revealed that undeclared synthetic drugs were found in herbal medicines and dietary supplements. The hidden drugs may cause serious toxic-effects to health. The adulterations of herbal medicine and food supplements are likely to contain indiscernible synthetic medicines, metals, or other toxic substances in high concentrations [7] and has become a problem all over the world. The “Adulteration of Chinese herbal medicines with synthetic drugs: a systematic review, Journal of Internal Medicine” (E. Ernst, 252 (2002) 107–113) showed that 24% of their 2600 Chinese herbal medicines samples contained at least one synthetic medicine [4], and 7% of the 260 Asian patient medicines (one component of TCM) from retail herbal stores in California that were tested were shown to contain an undeclared pharmaceutical [8]. Other than dietary supplements and sexual enhancement remedies, many other traditional herbal products were reported to be adulterated with various types of hidden synthetic chemicals capable of pharmacological activities [2]. These chemicals include the following: steroids (strength enhancers), nonsteroidal anti-inflammatory drugs, PDE-5 inhibitors or their analogues (sexual performance enhancers), antihypertensive agents, sibutramine and its analogues (weight loss products)[9], and many other types of therapeutic synthetic agents [10]. Currently, illegal drug detection in Taiwan is also conducted in accordance with the “Method of Test for Adulterants in Chinese Medicine and Foods,” which is the official inspection protocol by the Taiwan Food and Drug Administration (TFDA) [11]. Therefore, the development of improved analytical methodologies for the detection of adulterants is critically important to protect public health so that the quality of TCM and food supplements can be better insured.

The detection processes of TCM ingredients are complicated and challenging. Several methods are used to determine undeclared adulterants in herbal medicine or food supplements, including the following: LC-PDA [12], GC-MS [13], GC-QQQMS [14], LC-MS [15], LC-MS/MS [12], UPLC-TOF/MS [16], Q-Orbitrap MS [17], NMR [18], X-ray powder diffractometry [19], TLC-image [20], TLC-SERS [21], ATR‑IR [22], and CE-MS [23]. The applicability of GC‑MS is determined by the volatility and thermal stability of analytes. A faster technique for GC‑MS was discovered for the detection of sildenafil, tadalafil, and vardenafil in food and herbal products. A GC‑MS method was also successfully used for the screening of 134 pharmaceuticals in patent medications in China. By searching the NIST Mass Spectral Library and comparing the retention times, one can instantly screen out the ingredients and quantify them at the same time [24].

As soon as an unknown substance is found, GC-MS is performed for further verification. The following three gases are commonly used as carriers in gas chromatography (GC): nitrogen (N_2_), hydrogen (H_2_), and helium (He). Most GC studies commonly use He as the carrier gas. Helium on planet earth is generally found in natural gases and radioactive decay and is a relatively rare-5.2 ppm by volume in the atmosphere [25]. In anticipation of a potential helium-shortage crisis in the future, the price of helium is becoming expensive and vagaries in supply are limited. Thus, we describe the use of hydrogen instead of helium as a carrier gas for the analysis of illegal, adulterated drugs in TCM and food supplements using gas chromatography-mass spectrometry with electron ionization. Illicit drugs are investigated with hydrogen and helium to manifest the utility of hydrogen in the detection of the adulterant.

## Materials and methods

### Samples

Samples were taken from drugstores and herbal medicine stores in China, as well as suppliers and manufacturers by the Health Department of the Tainan County Government in Taiwan, between January 2015 and August 2016. The samples included 83 TCM samples and 40 food supplement samples (Please see S1 Table for sample details including sample name, description of appearance, purchase location and source origin).

### Chemicals and solutions

One hundred seventy pharmaceutical standards (purity ≥95%) were purchased from USP, TLC, Sigma-Aldrich, Cerilliant, European Pharmacopoeia, TCI, AK Scientific, AApin and Fluka (Please see S2 Table for standard details including compound name, molecular Weight, purity, brand, lot number, storage and source origin). Individual stock solutions (1,000 mg/L) were prepared by the dissolution of 10 mg of each compound in 10 mL of methanol, which were stored at -18°C. The mixed standard solutions at concentrations of 100 mg/L of each standard were prepared by the additive mixing 1 mL of each stock solution, and diluting it to 10 mL with methanol, respectively. HPLC grade methanol and ethanol were obtained from Merck (Darmstadt, Germany).

### Sample preparation

Five grams of sample were dissolved in 15–20 mL of ethanol and homogenized in an ultrasonic shaker for 30 minutes, followed by centrifugation at 3000 g for 5 minutes. The supernatant was filtered through a 0.22-μm PTFE syringe filter prior to being injected into GC-MS.

### GC-MS analysis

The analysis was performed on an Agilent 7890B GC system coupled to a 5977A MSD mass spectrometer (Agilent Technologies, Santa Clara, CA, USA) and equipped with a Gerstel Multipurpose sampler (Gerstel, Mülheim an der Ruhr, Germany). For the experiments using helium and hydrogen as a buffer gas, a Peak Precision Hydrogen Trace 500cc generator (Peak Scientific Instruments, Inchinnan, Scotland, UK) was used to generate the hydrogen gas. A silica capillary column, Agilent HP-5MS (30 m x 0.25 mm i.d. 0.25 μm film thickness), was used. The operation conditions were described in Table 1. The compounds’ spectra that were obtained were compared to the spectra of known compounds using the NIST Mass Spectral Search Program for the NIST/EPA/NIH Mass Spectral Library.

**Table 1:** Gas chromatograph-mass spectrometer settings.

Parameter	Setting	
Injector		
Carrier gas	Helium	Hydrogen
Flow	1.4 mL/min	1.0 mL/min
Pressure	9.4 psi	1.5 psi
Injection mode	Splitless mode	
Injection volume	1 μL	
Injector temperature	250°C	
Oven temperature program		
Initial ramp	80°C at 6°C/min until reaching 120°C	
Final ramp	At 8°C/min until reaching 300°C for 29 min.	
Mass spectrometer		
Ionization mode	Electron ionization	
Acquisition mode	Selected Ion Monitoring (SIM)	
Dwell time	100 ms	
Source temperature	230°C	
Quadrupole temperature	150°C	

### The limit of detection (LOD)

The experiment uses helium as a carrier gas to determine the monitored ion and retention time at 10 μg/mL. If the signal is too low to be detected, we increase the concentration of the drug standard gradually to 100, 500 and 1000 μg/mL until it can be obtained. The same experiment was performed for the comparison using hydrogen as the carrier gas for GC-MS.

Five grams of blank sample powder (Xiao Chai Hu Tong Extract Powder, Sheng Chang, Taipei, Taiwan) was spiked with the abovementioned concentration of drug standard (see 2.5.1) and dissolved in 15–20 mL of ethanol. A sample tube was homogenized in the ultrasonic shaker for 30 minutes and was followed by centrifugation at 3000 g for 5 minutes. The supernatant was filtered through a 0.22-μm PTFE syringe filter prior to being injected into the GC-MS. For the calculation of the method’s LODs, the fortification of three blank samples was performed in a specific concentration of drug standard. The concentration of the standard solution of which the ratio of peak height to noise was over 3 was defined as the LOD.

### Proficiency testing TFDA

We have conducted the Proficiency testing using the TFDA standards for the detection of drug-adulterants in traditional Chinese medicine in 2014 and 2015 through the use of hydrogen as a carrier gas by GC–MS. Proficiency testing is another effective tool that can be used to ensure the accuracy and precision of the results when using hydrogen as a carrier gas for the detection of adulterated drugs in Chinese herbal medicine and dietary supplements by GC–MS.

## Results

### GC-MS analysis

Each drug standard was analyzed with GC using a split-less injection to provide the MS identification of each compound and establish their retention time, LOD and identification ion in both helium and hydrogen as carriers in gas. All the results were obtained using the conditions described in Table 1. According to these results shown in Table 2, the use of hydrogen shortened the analysis time and saved resources. The optimum linear velocity for hydrogen is approximately 40 cm/s, which is about half of that of helium, which leads to a decrease in the analysis time by a factor of four, therefore making it possible to reduce the costs of analysis (less degradation of the capillary columns means a cheaper carrier gas). It is clear that the change from helium to hydrogen as the carrier reduced the run-times in drug analytes. Therefore, according to these results, the use of hydrogen allows for the acceleration of the analysis. The previous study revealed that it should be possible to reduce the length of the capillary column in order to save time and money without a loss of resolution in comparison to a longer column with helium as the carrier [26].

**Table 2:** Comparison retention time, signal-to-noise ratio(S/N) and monitored ion of drugs when hydrogen or helium is used as the carrier gas.

Drug	Carrier gas	Retention time (min)	S/N	Monitored ion (m/z)
Acetaminophen	H_2_	16.227	562.29	151, 109, 80, 83*
	He	17.045	415.15	109, 151, 43*, 80
Acetildenafil	H_2_	41.707	529.77	127, 70, 84, 42, 112, 56, 98
	He	44.943	159.15	127, 70, 84, 42, 112, 56, 98
Acetohexamide	H_2_	19.500	177.45	199, 184, 120*, 104, 91, 76, 64*, 51*
	He	20.254	238.09	184, 199, 76, 121*, 43*, 139*, 91, 104
Allopurinol	H_2_	22.872	797.93	136, 52, 109, 120, 67
	He	20.159	18.05	136, 52, 29*, 109, 67,120
Aminopyrine	H_2_	19.323	2492.34	231, 97, 77, 56
	He	20.163	3188.28	231, 56, 97, 77
Aminotadalafil	H_2_	46.301	131.81	390, 262, 204, 289, 233, 102, 169, 375, 43*, 405*
	He	49.941	133.16	390, 204, 262, 289, 169, 233, 115*, 169, 102, 375
Amitripthyline	H_2_	22.689	71.28	277, 202, 178, 152, 115, 91, 58
	He	23.578	144.76	277, 58, 202, 178, 115, 91, 152
Amphetamine	H_2_	6.278	84.52	44, 91,65,120
	He	6.103	575.81	44, 91,65,120
Aspirin	H_2_	8.926	138.22	120, 92, 152*, 65, 45*
	He	9.762	80.64	92, 120, 138*, 64*, 65
Atenolol	H_2_	25.113	12505.57	222, 107, 72
	He	25.693	3581.62	72, 107, 222
Atropine	H_2_	22.698	1471.07	124, 82, 94, 289, 140, 67, 103,42
	He	23.598	1486.90	124, 82, 94, 289, 140, 67, 42, 103
Barbital	H_2_	13.361	204.84	156, 141, 98, 112, 55, 41, 83, 69
	He	14.160	776.86	156, 141, 98, 55, 112, 41, 83, 69
**Benzbromarone**	H_2_	**28.290**	**204.84**	264, 173, 279, 115, 249, 328, 145, 132*, 221
	He	**29.169**	**6448.86**	264, 173, 115, 279, 249, 328, 145, 63*, 221
Benzocaine	H_2_	14.195	302.25	165, 120, 92, 65
	He	15.094	184.07	120, 165, 92, 65
Betamethasone	H_2_	29.912	273.45	312, 281*, 207, 160, 122, 91, 55
	He	30.794	381.89	122, 312, 91, 160, 207, 41, 55, 77*
**Bezafibrate**	H_2_	**26.950**	**6036.33**	120, 139, 107, 77*, 156
	He	**27.690**	**20.97**	120, 139, 205*, 107, 156
Bisacodyl	H_2_	29.089	23138.37	361, 319, 276, 246*, 199, 154
	He	29.996	18519.59	361, 276, 277*, 199, 319, 43*, 318*, 183*, 278*, 154
Bromhexine	H_2_	24.672	240565.29	376, 293, 264, 112, 70, 374*
	He	25.592	1374605.79	376, 293, 264, 305*, 112, 70
Brompheniramine	H_2_	21.654	1070326.01	247, 58, 167, 72, 180, 42, 139
	He	22.569	572.47	247, 58, 167, 72, 180, 42, 139
Bromvalerylurea	H_2_	12.902	18.40	137, 44, 100, 55, 83, 69, 120*
	He	13.809	43670.57	137, 44, 83, 180*, 100, 55, 69
Bucetin	H_2_	20.613	1837.10	223, 137, 108, 81, 53*
	He	21.466	1406.13	137, 108, 223, 45*, 81
Caffeine	H_2_	18.055	165.90	194, 109, 67
	He	18.878	283.12	194, 109, 67, 55*, 82*
Carbetapentane	H_2_	23.311	1995.44	86, 144, 115, 100, 58
	He	24.158	4056.80	86, 144, 115, 100, 58
Carbimazole	H_2_	15.606	417.25	186, 114, 72, 81, 42, 56, 127,141
	He	16.519	1395.15	186, 114, 72, 81, 141, 42, 56, 127
Carbinoxamine	H_2_	21.362	124.32	201, 167, 139*, 71
	He	22.248	181.70	201, 167, 58*, 71
Carbodenafil	H_2_	43.793	249.58	84, 56, 70, 381, 452, 339, 311, 42, 113, 136*
	He	47.202	96.80	84, 97*, 56, 70, 381, 452, 339, 311, 42, 113
Carisoprodol	H_2_	18.770	36.02	245, 184, 158, 97, 83*, 69*, 55
	He	19.689	20.54	245, 158, 97, 184, 55, 58*, 43*
Chloramphenicol	H_2_	27.569	2199.59	207, 172,153, 106, 77
	He	28.303	1989.30	207, 153, 172, 77, 106
Chlordiazepoxide	H_2_	29.238	170.17	282, 247, 220, 190, 165, 124*, 91
	He	30.310	246.65	282, 241*, 247, 220, 165, 190, 91
Chlormezanone	H_2_	23.419	3689.22	208, 174, 152, 125, 98, 69
	He	24.275	364.87	152, 208, 174, 98, 125, 69
Chlorpheniramine	H_2_	20.445	377.13	203, 58, 167, 72, 180, 42
	He	21.356	660.96	203, 58, 167, 72, 180, 42
Chlorpromazine	H_2_	25.943	393651.65	318, 272, 232, 196, 86, 58
	He	26.863	1034722.73	318, 58, 86, 272, 232, 196
Chlorpropamide	H_2_	16.426	413.58	190, 174, 127, 111, 75
	He	17.211	334.36	190, 111, 174, 127, 75
Chlorzoxazone	H_2_	16.681	1126.98	169, 113, 78
	He	17.724	428.48	169, 113, 78
Cimetidine	H_2_	5.722	101.59	45, 116, 55, 70, 60, 74, 42, 88*
	He	6.651	305.40	116, 45, 55, 60, 70, 74, 42, 99*
Cinnarizine	H_2_	31.221	640.13	201, 117, 167, 251, 152, 91
	He	32.229	1610.46	201, 117, 167, 251, 152, 91
Clobenzorex	H_2_	19.892	568.16	168, 127, 91, 65
	He	20.798	829.80	168, 127, 91, 65
Clofibrate	H_2_	13.722	657.00	242, 169, 128
	He	14.588	1238.64	128, 242, 169
Cocaine	H_2_	22.792	28.65	182, 82, 303, 105, 272, 198, 122, 51
	He	23.671	1156.47	182, 82, 303, 105, 272, 198, 122, 51
Colchicine	H_2_	33.875	133.19	399, 371, 312, 281, 254*
	He	35.326	126.04	312, 399, 371, 297*, 281
Cortisone	H_2_	27.709	131.20	122, 300, 91, 256, 105, 77, 147, 161, 55
	He	28.650	328.98	122, 300, 91, 256, 161, 147, 105, 55, 77
7-keto-DHEA	H_2_	28.240	616.13	302, 161, 91, 79, 105, 134, 187, 55, 41, 205
	He	29.196	3160.69	302, 161, 91, 79, 105, 134, 187, 55, 41, 205
N-Desmethylsibutramine	H_2_	18.139	8921.42	100, 58, 44, 137, 128, 115
	He	18.993	57424.38	100, 58, 44, 137, 128, 115
N-Didesmethylsibutramine	H_2_	18.111	4950.37	137, 115, 86
	He	18.989	2657.67	86, 137, 115
Dexamethasone	H_2_	29.940	336.90	312, 160, 122, 91, 55
	He	30.655	478.25	122, 312, 160, 91, 55
Dextromethorphan	H_2_	22.049	684403.48	271, 150, 214, 59, 171, 203, 128
	He	23.199	601.70	271, 59, 150, 214, 171, 203, 128
Diazepam	H_2_	25.356	191.73	283, 256, 221, 165, 77, 51
	He	26.238	790.25	283, 256, 221, 165, 77, 51
Dibucaine	H_2_	27.963	325.46	116, 86, 58
	He	28.855	689.64	116, 86, 58
Diclofenac	H_2_	21.958	530.12	295, 242, 214, 179, 151
	He	22.816	979.97	214, 295, 242, 179, 151
Dicyclomine	H_2_	21.505	853.46	86, 55
	He	22.375	5338.38	86, 99*, 55
Diethylpropion	H_2_	12.933	254.89	100, 77, 51
	He	13.797	995.63	100, 77, 51
Diethylstilbestrol	H_2_	23.917	2535.31	268, 239, 145, 107
	He	24.749	681.06	268, 107, 239, 145
Dimethylsildenafil	H_2_	45.707	227.53	113, 312, 70, 84, 42, 283, 136
	He	49.312	34.59	113, 312, 70, 84, 42, 283, 136
Diphenhydramine	H_2_	18.661	728.26	165, 58
	He	19.511	1728.75	165, 58
Diphenylhydantoin	H_2_	24.361	52.21	180, 223, 209, 252, 104, 77, 165, 147, 51
	He	25.460	47.80	180, 104, 223, 209, 252, 77, 165, 51, 147
Diprophylline	H_2_	24.230	165.79	254, 223, 180, 137, 109, 81, 54
	He	25.116	677.65	223, 254, 180, 109, 137, 81, 54
Econazole	H_2_	29.123	312.59	299, 207, 125, 81, 54
	He	30.013	73.59	125, 81, 299, 207, 54
**Estradiol benzoate**	H_2_	**35.659**	**4236.99**	**376, 105, 77**
	He	**37.513**	**1810.32**	**376, 105, 77**
Estriol	H_2_	29.054	707.09	288, 160, 146, 213, 133, 172, 201, 115, 185
	He	30.055	124.67	288, 160, 146, 213, 133, 172, 201, 115, 185
Estrone	H_2_	26.785	108.53	270, 146, 185, 213
	He	27.759	247.64	270, 146, 185, 213
Ethinylestradiol	H_2_	27.594	109.99	213, 296, 160, 133, 145, 228, 172, 185, 115
	He	28.434	146.01	213, 296, 160, 133, 228, 145, 172, 185, 115
Ethisterone	H_2_	27.721	21.79	124, 312, 91, 229, 245, 79, 105, 148, 286, 189, 67
	He	28.612	78.02	124, 312, 91, 79, 229, 245, 105, 148, 189, 67, 286
Ethoxybenzamide	H_2_	14.651	1578.34	165, 150, 120, 92, 65
	He	15.526	1168.66	120, 92, 150, 165, 65
Ethylestrenol	H_2_	24.277	3402.39	216, 241, 201, 288, 91, 270, 79, 121, 147, 105
	He	25.155	1896.52	216, 201, 241, 91, 79, 288, 270, 121, 147, 105
Fenfluramine	H_2_	7.506	4201.59	159, 109, 72, 56*
	He	8.267	8752.19	72, 159, 109, 44*
Finasteride	H_2_	33.484	208.98	372, 110, 58, 272, 357, 258, 128, 230, 72, 245
	He	34.822	168.39	372, 58, 110, 272, 357, 258, 128, 230, 72, 245
Flavoxate	H_2_	32.588	218.77	263, 234, 147, 98
	He	33.827	681.91	98, 234, 147, 263
Fluoxetine	H_2_	18.599	4320.42	309, 183, 162, 133, 104, 78*, 59
	He	19.489	10859.57	309, 183, 162, 133, 44*, 104, 59
Fluoxymesterone	H_2_	29.797	83968.78	336, 279, 109, 71
	He	30.742	145899.76	336, 279, 71, 109
Gemfibrozil	H_2_	19.329	1655.20	250, 122
	He	20.183	1357.76	250, 122
Gendenafil	H_2_	31.414	520.43	354, 326, 339, 136, 166, 282, 43, 311, 297
	He	31.414	514.07	354, 326, 339, 136, 166, 282, 43, 311, 297
Griseofulvin	H_2_	28.131	1108.72	352, 310, 284, 254, 214, 171, 138, 95, 69
	He	28.949	3255.28	352, 310, 138, 214, 284, 254, 69, 171, 95
Guaifenesin	H_2_	15.537	1687.05	124,109,198,77,95,65,52,167,149
	He	16.375	3642.70	124, 109, 198, 77, 81, 95, 65, 52, 167
Homatropine	H_2_	21.349	142.55	275, 124, 79
	He	22.276	514.41	124, 275, 79
Homosildenafil	H_2_	48.785	451.37	113, 70, 281*, 56, 42, 207, 355, 341, 309, 253
	He	53.164	463.73	113, 404*, 70, 56, 42, 207, 355, 341, 309, 253
Hydralazine	H_2_	19.201	343.84	160, 103, 131, 115, 89, 76, 145, 63, 50
	He	18.044	408.02	160, 103, 131, 115, 89, 76, 145, 63, 50
Hydrocortisone	H_2_	29.064	187.65	305*, 163, 123, 91, 55
	He	29.931	458.36	285*, 362*, 163, 123, 91, 55
Ibuprofen	H_2_	15.040	1090.68	206, 161, 117, 91, 65
	He	15.817	674.69	161, 206, 117, 91, 65
Imidazosagatriazinone	H_2_	27.187	9066.54	312, 284, 136, 240
	He	27.969	4361.89	312, 284, 136, 240
Indomethacin	H_2_	30.571	1204.53	139, 313*, 111, 75
	He	27.765	24.57	139, 357*, 111, 75
Ketoprofen	H_2_	22.913	54.02	105, 177, 209, 77, 254, 45*, 194, 131, 165
	He	23.701	158.29	105, 77, 177, 209, 254, 51*, 194, 131, 165
Lidocaine	H_2_	18.667	745.66	234, 120, 86, 58
	He	19.537	2480.92	86, 234, 120, 58
Lorazepam	H_2_	25.073	80.39	239, 274, 302, 75, 138, 177, 111, 203, 163, 100
	He	25.916	448.96	239, 274, 302, 75, 138, 177, 111, 203, 163, 100
Mazindol	H_2_	24.604	3833.98	266, 231, 204, 176, 128, 102, 75
	He	25.835	382.97	266, 231, 204, 176, 128, 102, 75
**Mefenamic acid**	H_2_	**22.179**	**2403.18**	241, 223, 180, 152, 102*
	He	**22.732**	**186.25**	223, 241, 180, 77*, 152
Melatonin	H_2_	24.765	855.84	232, 172, 160, 145, 130, 117, 102, 89
	He	25.586	834.77	160, 172, 232, 145, 117, 130, 102, 89
Mephenesin	H_2_	14.300	44.99	182, 108, 91
	He	15.027	2776.31	108, 182, 91
Mephentermine	H_2_	9.224	3957.33	72, 91, 148, 56, 42, 115
	He	8.883	1232.24	72, 91, 148, 56, 42, 115
Meprobamate	H_2_	17.741	904.61	83, 55, 43, 71, 62, 96, 114, 144, 101
	He	18.516	2359.60	83, 55, 71, 62, 96, 114, 144, 101, 43
Methamphetamine	H_2_	6.505	2216.40	58, 91, 65, 134, 42, 115, 119*
	He	7.195	3184.90	58, 91, 65, 56*, 134, 42, 115
Methandriol	H_2_	26.350	74.00	253, 213, 271, 304, 105, 145, 286, 228, 119, 159
	He	27.230	305.46	253, 213, 304, 105, 145, 271, 286, 228, 119, 159
Methandrostenolone	H_2_	27.749	76.18	122, 91, 161, 147, 105, 134, 77
	He	28.686	360.69	122, 91, 161, 147, 105, 134, 77
Methaqualone	H_2_	22.282	1208.48	250, 91, 132, 65, 77, 217, 117, 50, 104*
	He	23.192	3143.27	235*, 250, 91, 132, 65, 77, 217, 117, 50
Metharbital	H_2_	12.212	2739.29	155, 112, 83, 55
	He	12.953	2922.10	155, 170*, 112, 83, 55
Methimazole	H_2_	13.837	140.23	114, 72, 81, 42, 54, 86, 59
	He	14.903	213.63	114, 72, 42, 81, 54, 86, 59
Methylprednisolone	H_2_	29.813	457.10	136, 91, 55*
	He	30.677	1796.27	136, 91, 121*
Methyltestosterone	H_2_	27.435	26731.78	302, 229, 202, 161, 124, 91
	He	28.384	1780.50	302, 124, 91, 229, 202, 161
Metoclopramide	H_2_	27.217	5295.57	184, 86, 58
	He	28.034	8438.34	86, 184, 58
Metronidazole	H_2_	15.394	443.85	171, 124, 81, 53
	He	16.174	423.83	124, 81, 171, 53
Minoxidil	H_2_	20.855	91.48	193, 164, 138, 110, 84, 67
	He	21.700	1697.28	193, 164, 110, 138, 84, 67
Morphine	H_2_	25.421	592.33	285, 162, 42, 215, 115, 55, 65, 92, 81
	He	26.311	2101.32	285, 162, 42, 215, 115, 55, 65, 92, 81
Nalidixic acid	H_2_	25.020	2964.06	188, 160, 132, 173, 145, 104, 232, 77
	He	25.602	4290.93	188, 160, 132, 173, 145, 104, 232, 77
Nandrolone	H_2_	25.593	112.00	274, 215, 173*, 147, 119, 91, 67
	He	27.554	413.47	274, 110*, 91, 67, 119, 215, 147
Naproxen	H_2_	21.384	479.13	230, 185, 170, 141, 115
	He	31.254	21.76	185, 230, 170, 141, 115
Nifedipine	H_2_	26.652	2198.09	329, 284, 224, 268, 254, 195, 180
	He	27.496	8691.50	329, 284, 224, 268, 254, 195, 180
Noracetildenafil	H_2_	39.756	143.98	113, 70, 42, 56, 98, 207, 311, 452, 136, 354
	He	42.433	144.95	113, 70, 42, 56, 98, 207, 452, 311, 136, 354
Norethisterone	H_2_	27.236	237.22	298, 283*, 265
	He	28.102	855.06	298, 231*, 265
Orphenadrine	H_2_	19.556	345.15	58, 73, 165, 178, 45
	He	20.419	1799.00	58, 73, 165, 178, 45
Oxethazaine	H_2_	26.450	198.46	114, 86, 213, 56, 133, 72, 304
	He	27.281	289.85	114, 86, 56, 213, 133, 72, 304
**Oxymetholone**	H_2_	**28.615**	**8872.16**	**174, 275, 332, 43, 161, 91, 81, 71, 216, 107**
	He	**29.471**	**16927.40**	**174, 332, 275, 216, 161, 107, 43, 91, 81, 71**
Oxyphenbutazone	H_2_	30.658	324.39	93, 45*, 55, 69, 161, 193*, 77, 249*
	He	31.419	312.73	93, 199*, 77, 324*, 55, 69, 161
Pentazocine	H_2_	23.647	451.43	217, 202, 285, 110, 270, 70, 45, 159, 173
	He	24.505	1600.48	217, 202, 110, 285, 270, 70, 45, 159, 173
Phenacetin	H_2_	16.184	2534.87	179, 137, 108, 80, 65, 53
	He	17.017	1248.32	108, 179, 137, 80, 65, 53
Phenazopyridine	H_2_	23.833	1046.17	213, 108, 81, 54, 136, 97*, 184, 66, 155
	He	24.670	660.83	213, 108, 81, 136, 54, 184, 66, 51*, 155
Phenformin	H_2_	14.011	274.32	146, 104, 91, 77, 65
	He	14.844	412.63	91, 146, 104, 77, 65
Phenobarbital	H_2_	20.141	3530.71	204, 117, 232, 161, 146, 103, 77, 91, 174
	He	20.996	2851.29	204, 117, 232, 161, 146, 77, 103, 91, 174
Phenolphthalein	H_2_	31.728	255.45	318, 274, 225, 181, 152, 104*, 65
	He	32.746	765.90	274, 318, 225, 181, 152, 121*, 65
Phentermine	H_2_	6.160	2271.97	70, 91, 105, 58, 65, 115, 41, 115
	He	6.779	3375.10	70, 91, 105, 58, 65, 115, 41, 115
Phentolamine	H_2_	26.987	42.57	199, 183, 91, 154, 77, 128, 170
	He	27.784	117.12	199, 183, 91, 154, 77, 128, 170
Phenylbutazone	H_2_	24.709	3051.14	308, 252, 183, 152, 105, 77
	He	25.543	5464.75	183, 308, 252, 77, 152, 105
Phenylephrine	H_2_	18.847	94.46	135, 44, 107, 179, 160, 77, 51, 91
	He	16.485	224.29	135, 44, 107, 179, 160, 77, 51, 91
Phenylpropanolamine	H_2_	9.731	236.24	44, 77, 105, 51, 117, 91
	He	10.797	1849.14	44, 77, 51, 105, 117, 91
**Piperidenafil**	H_2_	**44.617**	**128.79**	**431, 459, 283, 67, 42, 84, 121, 135, 149, 215**
	He	**48.328**	**21.36**	**431, 459, 283, 67, 42, 84, 121, 135, 149, 215**
Pirenzepine	H_2_	31.102	253.75	351, 281, 211, 113, 70
	He	32.009	358.14	113, 70, 211, 351, 281
Piroxicam	H_2_	16.877	1640.49	104, 76, 43, 152, 169, 118, 386*, 211, 91
	He	17.665	652.90	104, 152, 76, 43, 169, 118, 211, 91
Prednisolone	H_2_	29.312	68.40	122, 91, 55
	He	30.225	1015.08	122, 91, 55
Prednisone	H_2_	27.889	417.77	298, 245, 226, 186, 160, 131, 115, 91
	He	28.785	534.87	298, 160, 91, 245, 226, 186, 131, 115
Primidone	H_2_	23.472	1344.44	146, 190, 117, 161, 103, 91, 77, 174
	He	24.067	1431.77	190, 146, 117, 161, 103, 91, 77, 174
Probenecid	H_2_	22.941	295.85	270, 135, 199, 104, 76, 43
	He	23.788	142.22	270, 135, 199, 104, 76, 43
Procaine	H_2_	20.737	341.47	86, 99, 120, 65, 56, 164
	He	21.644	523.45	86, 99, 120, 164, 65, 56
Progesterone	H_2_	28.663	54.72	314, 272, 229, 147, 124, 91, 67
	He	29.599	358.43	124, 314, 272, 229, 91, 67, 147
Propantheline	H_2_	24.358	735.98	86, 181, 310, 99, 152, 44, 58, 325, 127, 71
	He	25.183	3169.32	86, 181, 44, 99, 310, 152, 58, 325, 127, 71
Propranolol	H_2_	22.279	745.35	259, 215, 144, 115, 72
	He	23.188	3138.57	72, 115, 144, 259, 215
Quinine	H_2_	28.967	2199.60	189, 160, 136
	He	29.839	4577.02	136, 189, 160
Ranitidine I	H_2_	21.701	272.14	235, 137, 94, 67
	He	22.514	497.94	137, 235, 94, 67
Ranitidine II	H_2_	28.971	279.25	
	He	29.960	1175.13	
Rimonabant	H_2_	35.656	601.19	84, 363, 55, 99, 282, 335, 299, 41, 380, 145, 462*
	He	37.392	1808.45	84, 55, 99, 363, 282, 335, 299, 41, 380, 111*, 145
Salicylamide	H_2_	12.103	1129.40	120, 137, 92, 65, 53, 44, 80
	He	12.979	457.91	120, 137, 92, 65, 53, 44, 80
Salicylic acid	H_2_	9.100	1859.82	120, 92, 138, 64, 46
	He	9.655	1343.64	120, 92, 138, 64, 46
Scopolamine	H_2_	24.172	1502.23	94, 138, 108, 154, 303
	He	25.121	3631.33	94, 138, 108, 154, 303
Secobarbital	H_2_	17.809	2691.55	195, 168, 124, 97, 53
	He	18.684	1204.76	168, 195, 97, 124, 53
Sibutramine	H_2_	18.276	16371.68	114, 72, 58, 101, 128, 137
	He	19.109	5010.93	114, 72, 58, 101, 128, 137
Sildenafil	H_2_	45.447	145.57	404, 281*, 207, 99, 56
	He	49.500	17.60	99, 404, 56, 283*, 207
Stanozolol	H_2_	31.003	163.95	96, 328, 257, 270, 133, 119, 175
	He	32.022	335.89	96, 328, 257, 270, 133, 119, 175
Strychnine	H_2_	31.979	2444.29	334, 167, 130, 107, 77, 55
	He	33.167	296.67	334, 167, 130, 107, 77, 55
Sulfadiazine	H_2_	26.540	512.14	185, 92, 65, 108
	He	27.429	756.37	185, 92, 65, 108
Sulfadimethoxine I	H_2_	28.890	219.77	259, 140, 92, 65, 168, 108, 121, 82, 187
	He	29.806	639.59	259, 140, 92, 65, 168, 108, 121, 82, 187
Sulfadimethoxine II	H_2_	29.269	364.43	
	He	30.114	208.79	
Sulfamerazine	H_2_	27.012	801.84	199, 92, 65
	He	27.910	1340.67	200*, 199, 92, 65
Sulfamethazine	H_2_	27.373	2275.83	213, 92, 65
	He	28.212	1074.36	214*, 213, 92, 65
Sulfamethoxazole	H_2_	25.579	1070.19	92, 108, 65, 156, 119, 162, 174, 43*, 140*
	He	26.156	910.57	92, 156, 108, 65, 162, 119, 253*, 174, 189*
Sulfamethoxypyridazine	H_2_	28.799	157.78	215, 92, 108, 65, 53, 80, 280
	He	29.673	214.35	215, 92, 65, 108, 53, 280, 80
Sulfanilamide	H_2_	20.112	263.20	172, 92, 65
	He	20.718	202.79	172, 92, 65, 108*
Sulfinpyrazone	H_2_	23.429	761.20	278, 249, 209, 183, 152, 130, 105, 77, 51
	He	24.242	1169.11	278, 77, 105, 130, 51, 249, 209, 183, 152
Sulindac	H_2_	29.207	198.68	233, 297, 312, 248, 67, 123, 47, 133, 220
	He	30.083	516.90	297, 233, 312, 248,123, 220, 67, 47, 133
Synephrine	H_2_	15.602	43.68	135*, 44, 107, 179*, 160*, 77, 51, 91
	He	16.281	35.97	44, 77, 108*, 107, 65*, 51, 91
Tadalafil	H_2_	41.729	1803.01	389, 262, 204, 169
	He	44.673	79.24	389, 262, 204, 169
Terbinafine	H_2_	23.292	289.12	141, 276, 234, 115, 291, 196
	He	24.139	1216.53	141, 276, 115, 234, 291, 196
Testosterone	H_2_	27.168	65.84	288, 246, 203, 147, 124, 91, 55
	He	28.115	328.53	124, 288, 246, 147, 203, 91, 55
Tetracaine	H_2_	23.189	5752.14	58, 71, 176, 150, 105, 193, 92
	He	24.012	3991.85	58, 71, 176, 150, 105, 193, 92
Theobromine	H_2_	19.022	23.33	180, 67, 109, 55, 82, 137, 42, 94
	He	19.157	142.02	180, 67, 55, 109, 82, 137, 42, 94
Theophylline	H_2_	20.324	33.21	180, 95, 68, 53
	He	21.141	246.52	180, 95, 68, 53
Thiodimethylsildenafil	H_2_	40.912	33.32	113, 70, 42*, 84, 328*, 343, 56
	He	43.571	48.61	113, 340*, 70, 84, 283*, 56, 343
Thiohomosildenafil	H_2_	50.969	202.81	113, 70, 56, 475, 98, 42, 327, 341, 269, 84
	He	55.952	201.35	113, 70, 56, 475, 98, 42, 327, 341, 269, 84
Thioridazine	H_2_	31.712	366.09	370, 244, 185, 126, 98, 70
	He	32.798	120.18	98, 370, 70, 185, 244, 126
Thiosildenafil	H_2_	47.756	296.39	99, 448, 56, 489, 425, 70, 207
	He	51.652	355.43	99, 448, 56, 70, 489, 425, 207
Tinidazole	H_2_	21.175	776.12	201, 123, 80, 68, 93, 107, 53, 154, 247
	He	21.921	3229.62	201, 53, 80, 123, 68, 93, 107, 154, 247
Tolbutamide	H_2_	15.612	426.74	91, 171, 155, 65, 107, 77, 197
	He	16.398	684.97	91, 171, 155, 65, 107, 77, 197
Vardenafil analogue	H_2_	27.628	5472.86	284, 312, 256, 67, 297, 120, 269, 93, 135
	He	28.462	2912.76	284, 312, 256, 297, 67, 120, 269, 93, 135
Yohimbine	H_2_	32.330	1464.36	353, 169
	He	33.427	1587.82	353, 169
Zolpidem	H_2_	28.784	378.38	235, 207, 219, 281*, 307, 65, 92, 191
	He	29.717	205.59	235, 307, 219, 92, 65, 191, 207
**Cetilistat**	H_2_	**13.943**	**218.91**	**177, 160, 133, 55, 104, 77, 401**
	He	**14.692**	**234.20**	**177, 160, 133, 104, 55, 77, 401**

*represent the differences between the H_2_ and He ions.

**Fig 1:**
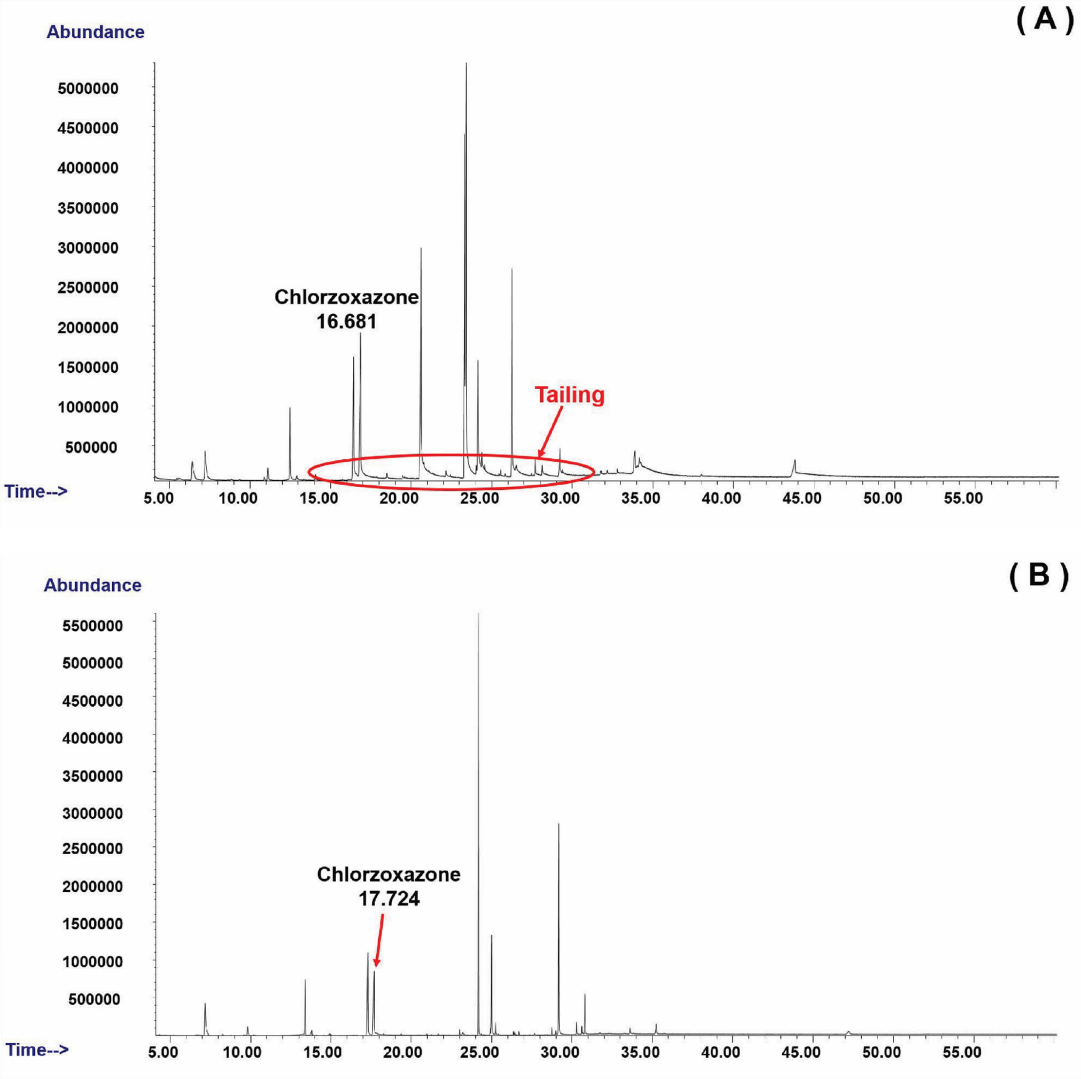
Ion chromatogram of chlorzoxazone when (A) hydrogen and (B) helium is used as a carrier gas.

**Fig 2:**
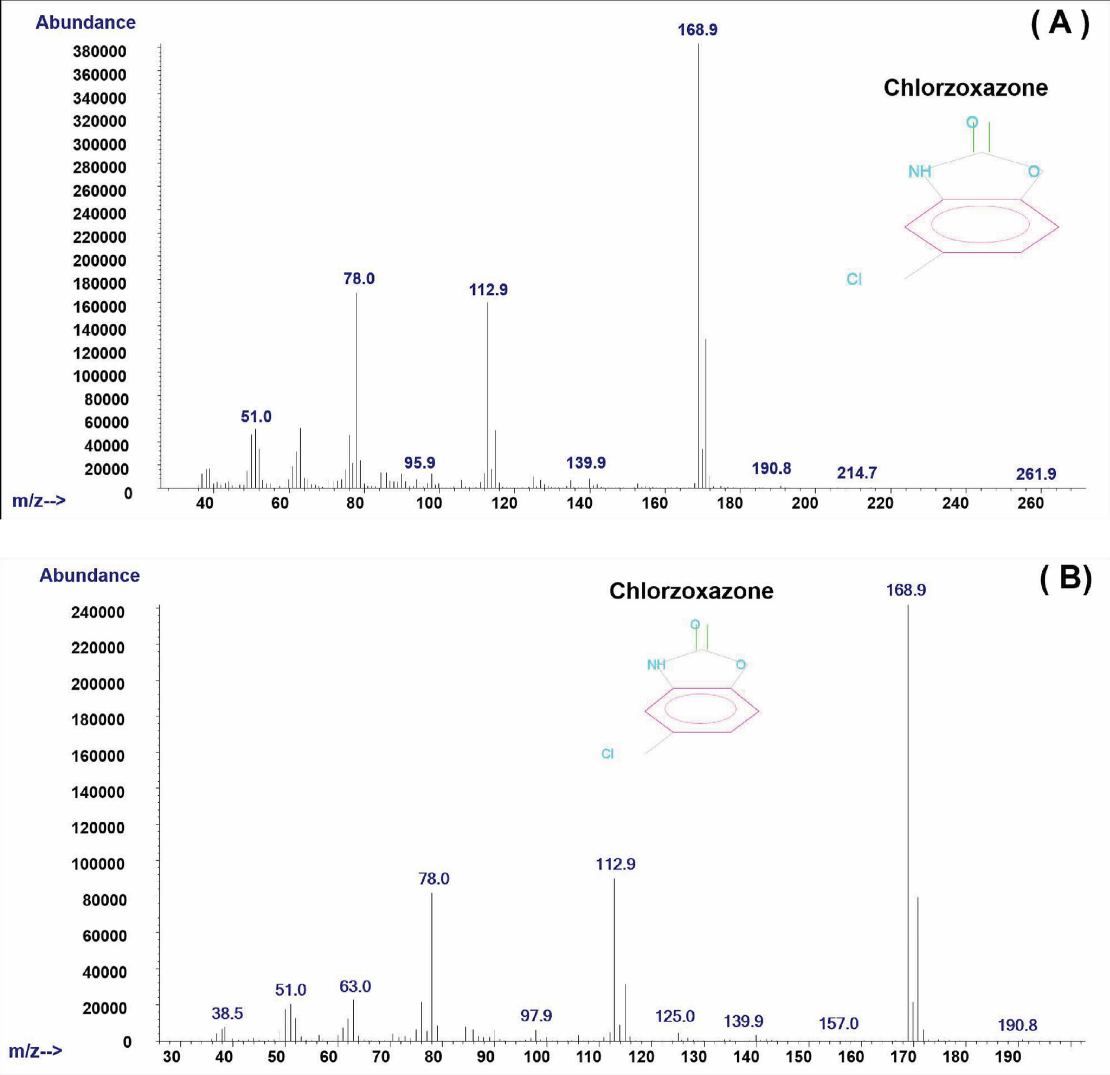
Mass spectrum of chlorzoxazone when (A) hydrogen and (B) helium is used as a carrier gas.

**Fig 3:**
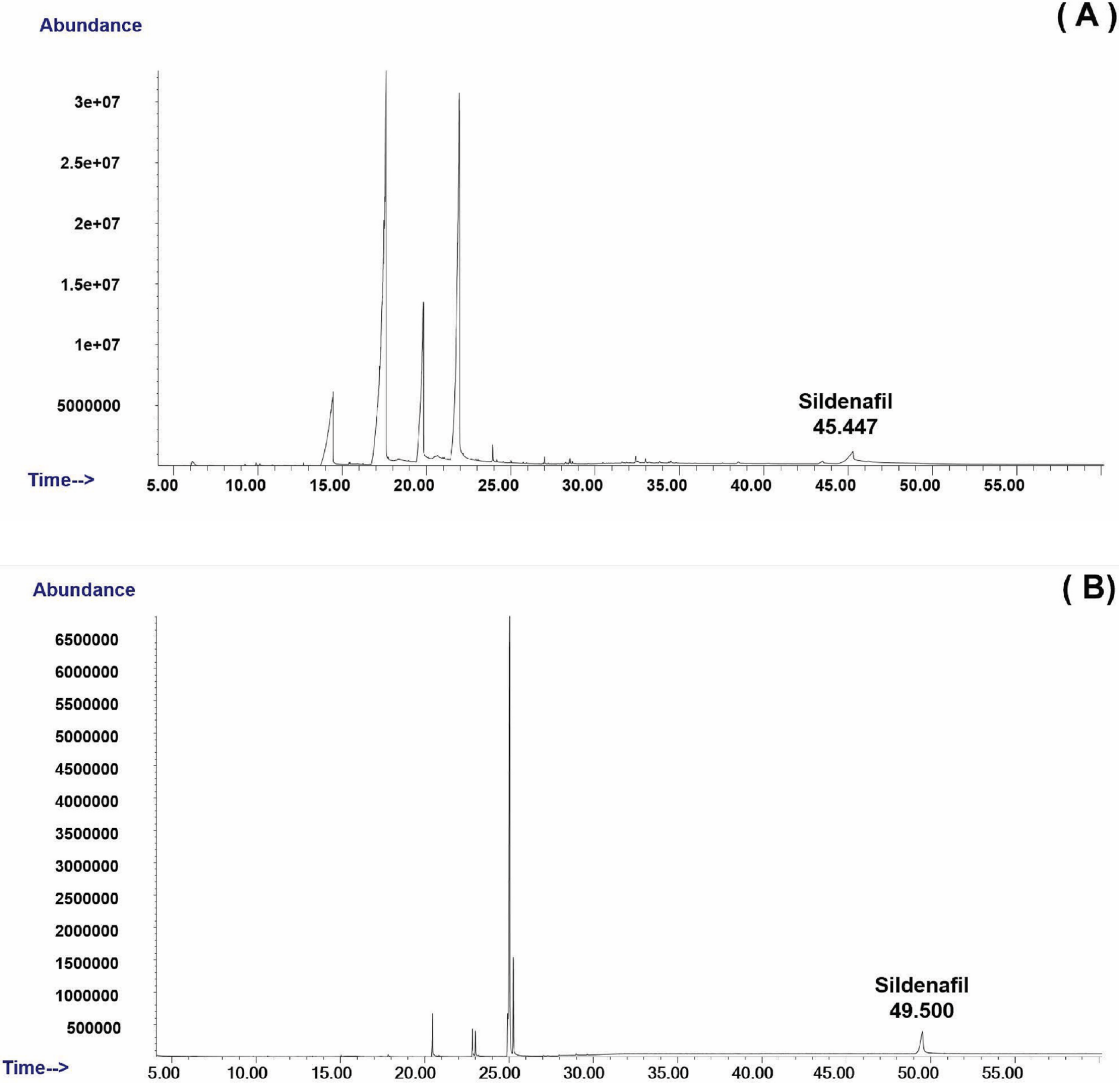
Ion chromatogram of sildenafil when (A) hydrogen and (B) helium is used as a carrier gas.

**Fig 4:**
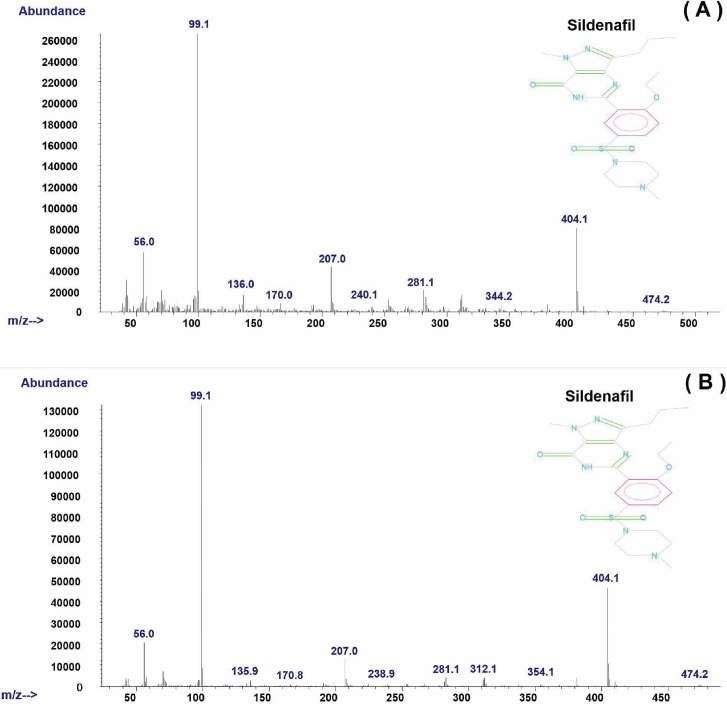
Mass spectrum of sildenafil when (A) hydrogen and (B) helium is used as a carrier gas.

Fig 1 presents the chromatograms of the chlorzoxazone obtained when hydrogen (A) and helium (B) were used as the carrier gas at the retention times of 16.681 and 17.724 min. It can be observed that the use of hydrogen allowed for a reduction in the run-time analysis of 1.043 min and led to a slight peak tailing. However, the same mass spectrum of chlorzoxazone was found by GC-MS when hydrogen (A) and helium (B) were used as the carrier gas in Fig 2. Fig 3 shows a comparison of the chromatograms of sildenafil with hydrogen (A) and helium (B) at the retention times of 45.447 and 49.500 min. An elimination of 4.053 min in the run-time analysis was observed with the use of hydrogen. Similar mass spectra of hydrogen (A) and helium (B) as the carrier in gas are shown in Fig 4.

### LOD

In considering the limit of detection (LOD), a signal-to-noise ratio of 3 was defined. The LODs of amitriptyline and 45 other analytes were 10 μg/g, and those of 104 analytes containing acetaminophen were 100 μg/g. The LOD of allopurinol was 500 μg/g, and those of the other 19 drugs—including aminotadalafil—were 1,000 μg/g. Moreover, the monitored ion library of the total analytes was developed for adulterant identification and monitoring. The 170 drugs obtain almost the same level of LODs with the use of hydrogen and helium as a carrier gas as detected by GC–MS for TCM and food supplements. Except for benzbromarone, estradiol benzoate, bezafibrate, mefenamic acid, oxymetholone, piperidenafil and cetilistat, the LODs of those analytes using hydrogen (100, 100, 1000, 1000, 1000, 1000 and 1000 μg/g, respectively) were decoupled as much as those of the previous analytes using helium (10, 10, 100, 100, 100, 100 and 100 μg/g, respectively).

### Proficiency testing TFDA

Proficiency testing is another effective tool that can be used to ensure the results using hydrogen as a carrier gas for the detection of adulterated drugs in TCM and dietary supplements by GC–MS. Using the abovementioned detection method performed in testing for Chinese herbal medicine adulteration held by the TFDA revealed satisfactory results in 2015 and 2016. The analytical results were obtained using qualitative analysis. This result also provided the opportunity for the present method to verify the performance in testing for Chinese herbal medicine adulteration.

### Analytical results of drugs in TCM and supplement foods on the market

In the present study, forty food supplement samples and eighty-three TCM samples were inspected and detected simultaneously with helium and hydrogen as a carrier gas by GC–MS between 2015 and 2016. Out of all 123 samples, 115 were found to be untainted, and the remaining 8 were TCM samples that were found to contain illegal, adulterated drugs (see in Table 3). The detected drug adulterants of 123 TCM and food supplement samples are shown in Table 4.

**Table 3:** Analytical results of TCM and food supplement samples.

Type of sample	TCM	Food supplement	Total
Number of samples	83	40	123
Number of samples detected	8	0	8
Positive rate (%)	9.6	0	6.5

**Table 4:** Detected drug frequency in TCM and food supplement samples.

Drugs	Detected frequency
Acetaminophen	3
Chlorzoxazone	3
Ibuprofen	3
Sulfamethoxazole	2
Sildenafil	2
Tadalafil	1

The types, forms and numbers of adulterated drugs in 8 TCM samples were 3 capsules, 3 powders, 1 paste and 1 medicated patch/plaster. The three capsule samples were detected with the kidney-supplement category of sildenafil or tadalafil. The three powder samples were all detected with the antirheumatic-analgesics category of acetaminophen, ibuprofen and chlorzoxazone. One paste and one medicated patch/plaster sample were detected with the antidote category of sulfamethoxazole. The analytical results were consistent with the use of hydrogen and helium as a carrier gas by GC-MS in the current study. In brief, the above evidence demonstrated the availability of the method with the use of hydrogen as a carrier gas for the detection of adulterated drugs in traditional Chinese medicine and dietary supplements using GC-MS for real sample analysis. The previous study also demonstrated the feasibility of using hydrogen as an alternative carrier gas, which has been in use for the routine analysis for government regulations for most estrogens, androgens and gestagens of the Belgium national plan [27].

## Discussion

Hydrogen is a highly effective carrier gas because it increases the speed of the analysis and the resolution in GC [28]. Hydrogen offers the chromatographer a number of benefits, including increased speed, lower temperature separations, longer column life, fewer environmental concerns and greater availability [29]. However, some safety concerns are associated with the use of hydrogen cylinders, such as the following: cylinder handling and storage, the flammable nature of hydrogen and the variation in quality of the gas selection of the appropriate gas delivery equipment to ensure the system’s purity. In addition, hydrogen is flammable over a wide concentration range in air from 4% to 74.2% by volume, it has the highest burning velocity of any gas, and it can self-ignite due to very low ignition energy when expanding rapidly from high pressure [27]. As an alternative to cylinders, hydrogen generators provide a continuous source of high purity hydrogen and can eliminate many safety concerns over using hydrogen cylinders. Because hydrogen can be generated on demand, the volume of stored gas in a hydrogen generator is very small. Moreover, it has built-in safety features. In the case of a leak, the flow of hydrogen will be automatically shut down to ensure that it never reaches to the lower explosive limit. In addition to safety concerns, reactions in the ion source, the loss of functionality of the pumping system, and high background noise are also disadvantages of using hydrogen as a carrier gas [30].

Hydrogen is a reactive gas, and it might react with analytes under certain conditions. The major adverse effect of hydrogen is on the GC injector liner activation, which can catalytically degrade samples such as certain simple pesticides [31]. Chromatographers should avoid the use of chlorinated solvents with the hydrogen carrier gas because of the risk of hydrochloric acid (HCl) formation, which can affect the performance of the chromatographic system. All situations should be carefully evaluated when changing to hydrogen as a carrier gas. Furthermore, the ability to switch from He to H_2_ as a carrier gas will save money and time.

Although hydrogen seems to be an ideal gas for GC/MS and it offers important advantages over helium in terms of efficiency, resolution and the speed of analysis, some disadvantages should be mentioned. The study suggested that hydrogen as a carrier gas had excellent performance that was comparable to using helium for the nonpolar, nonreactive compounds. Thus, the most polar, reactive compounds displayed significantly lower responses with the hydrogen carrier gas. Furthermore, evidence demonstrated that nitrobenzene, which is one of the most reactive compounds, was reduced to aniline when using hydrogen as a carrier [32]. Hydrogen is a reactive compound that might hydrogenate unsaturated and aromatic compounds under certain conditions. Hydrogen reduces metal oxides at the ion source and exposes bare and highly active metal surfaces at the EI (and CI) ion source. Thus, many compounds are degraded at the ion source, lose their molecular ions and are harder to identify by the library [31]. Previous experiments showed that the baseline of the total ion chromatograms is elevated in hydrogen relative to that in helium. The obtained signal-to-noise ratio is poorer with hydrogen compared with helium via both a lower signal and higher noise. The S/N values are approximately 3-5-fold lower when hydrogen is used as a carrier gas compared to the results using helium. Decreased response factors for some analytes may result from chemical interactions with hydrogen in the MS ion source or other causes [32]. The lower signal-to-noise ratio of hydrogen might lead to the lower LOD in a certain compound. Among the 170 analytes studied, 7 drugs using hydrogen as the carrier gas provided an LOD ten times poorer than those using helium as the carrier gas. The same LOD was found for all other 163 drugs. The 7 drugs were benzbromarone, estradiol benzoate, bezafibrate, mefenamic acid, oxymetholone, piperidenafil and cetilistat. [33]. The structure suggested the potential reactivity of the 7 analytes with the hydrogen carrier gas. Therefore, recent work has reported on the unstable signal and reduced accuracies of the pesticides when hydrogen was used as carrier gas, and moreover some compounds were undetectable rather than those when helium was used as a carrier gas [34]. The research indicated that fragmentation patterns are similar whether helium or hydrogen is used as the carrier gas except nitrobenzene. Nitrobenzene can be reduced in the presence of hydrogen, thus resulting in the different fragmentation patterns and peak tailings [35]. Moreover, in general, more abundant fragmentation is observed, and higher relative abundances of the diagnostic ions for the identification of the components using hydrogen as a carrier gas. In the case of Py-GC, using H_2_ as a carrier gas may cause unwanted protonation or hydrogenation reactions, which may lead to difficulties in the library search when using existing MS libraries [36]. One must carefully consider the chemistry of specific analytes when changing to hydrogen as a carrier gas. The potential reactivity of analytes with the hydrogen carrier gas should be evaluated in the early stages of the method’s development.

In this study, an economical analytical method for the determination of adulterated drugs in traditional Chinese medicine and dietary supplements with GC-MS using hydrogen as a carrier gas was developed. In general, helium is considered to be the most widely used carrier gas for GC–MS analysis. However, the cost of helium is increasingly expensive due to its limited supply. Hydrogen is as an alternative GC-MS carrier gas. The hydrogen generator produces ultrahigh purity hydrogen through the electrolysis of deionized-distill water without cost and usage limits; moreover, there were no safety concerns associated with high pressure cylinders. Hydrogen is not only renewable, abundant and economical, but it also offers important advantages in terms of reduced run-times and performance benefits over helium [27]. The screening of all TCM and dietary supplements proved to be necessary for the detection of pharmaceutical substances to protect consumers from adverse reactions and side effects before the products are made available on the market. The screening of illegal drug adulteration using hydrogen instead of helium as a carrier gas has been in use for routine analysis in our laboratory for 2 years now. Eight products adulterated with acetaminophen, ibuprofen, chlorzoxazone, sulfamethoxazole, tadalafil and sildenafil substances that are prohibited in TCM were detected as the result of 123 TCM and food supplements’ screening. Satisfactory consistency between the hydrogen and helium spectra of illicit drugs in real sample analysis also demonstrates that hydrogen can be used effectively as a buffer gas in GC-MS.

## Supporting information

S1 Table Information of samples. (DOCX)

S2 Table Information of standard. (DOCX)
