# Supplementary material for: Detection of adulterated drugs in traditional Chinese medicine and dietary supplements using hydrogen as a carrier gas
Source: PLoS One. 2018 Oct 10;13(10):e0205371. doi: 10.1371/journal.pone.0205371 (PMC6179279; doi:10.1371/journal.pone.0205371)
Supplement: S1 Table — (DOCX) [file pone.0205371.s001.docx]

**S1 Table. Information of Samples**

| **Sample Number** | **Sample Name** | **Description of appearance** | **Purchase locations** | **Source origin** |
| --- | --- | --- | --- | --- |
| 1 | Lutein Complex Capsules | Capsule | No.5-11, Siajhaizih, Sigang Dist., Tainan 72345, Taiwan | Xiang Yi Biotech co LTD |
| 2 | Liquid medicine | Liquid medicine | No.18, Jiouru 1st Rd., Sanmin Dist., Kaohsiung 80766, Taiwan | No.18, Jiouru 1st Rd., Sanmin Dist., Kaohsiung 80766, Taiwan |
| 3 | Zishen Pill | Chinese medicine pill | No.385-1, Sec.2, Jhongjheng Rd., Rende Dist., Tainan 71743, Taiwan | No.385-1, Sec.2, Jhongjheng Rd., Rende Dist., Tainan 71743, Taiwan |
| 4 | Chinese medicine pill(black) | Chinese medicine pill (black) | No.263, Fusing Rd., Sinying Dist., Tainan 73043, Taiwan | No.263, Fusing Rd., Sinying Dist., Tainan 73043, Taiwan |
| 5 | Chinese medicine powder | Chinese medicine powder | No.263, Fusing Rd., Sinying Dist., Tainan 73043, Taiwan | No.263, Fusing Rd., Sinying Dist., Tainan 73043, Taiwan |
| 6 | Haishaodan | Chinese medicine pill | No.263, Fusing Rd., Sinying Dist., Tainan 73043, Taiwan | No.263, Fusing Rd., Sinying Dist., Tainan 73043, Taiwan |
| 7 | Zuogui pill | Chinese medicine pill | No.263, Fusing Rd., Sinying Dist., Tainan 73043, Taiwan | No.263, Fusing Rd., Sinying Dist., Tainan 73043, Taiwan |
| 8 | Capsule(blue) | Capsule (blue) | No.307, Guantian, Guantian Dist., Tainan 72047, Taiwan | No.307, Guantian, Guantian Dist., Tainan 72047, Taiwan |
| 9 | Chinese medicine powder | Chinese medicine powder | No.23, Dongciao 10th St., Yongkang Dist., Tainan 71048, Taiwan | No.23, Dongciao 10th St., Yongkang Dist., Tainan 71048, Taiwan |
| 10 | Chinese medicine powder | Chinese medicine powder | No.187, Sec.1, Beian Rd., Bei Dist., Tainan 70465, Taiwan | No.187, Sec.1, Beian Rd., Bei Dist., Tainan 70465, Taiwan |
| 11 | Bamboo Charcoal Ginseng Healthy Patch | Patch | No.10-9, Madoukou, Madou Dist., Tainan 72154, Taiwan | No.10-9, Madoukou, Madou Dist., Tainan 72154, Taiwan |
| 12 | Capsules | Capsule | No.35, Meihe St., Mituo Dist., Kaohsiung 82743, Taiwan | No.35, Meihe St., Mituo Dist., Kaohsiung 82743, Taiwan |
| 13 | SunsenGinbo Capsules | Capsule | No.99-6, Nanmen Rd., Jhongsi Dist., Tainan 70049, Taiwan | 6F., No.352, Fumin Rd., Zuoying Dist., Kaohsiung 81358, Taiwan |
| 14 | Ginsenoside | Capsule | No.676, Chongde Rd., Dong Dist., Tainan 70173, Taiwan | Hsinten International Group 7F., No.217, Sec.2, Sinbei Blvd., Sinjhuang Dist., New Taipei City 24250, Taiwan |
| 15 | SOQI Capsules | Powder | No.676, Chongde Rd., Dong Dist., Tainan 70173, Taiwan | Hsinten International Group 7F., No.217, Sec.2, Sinbei Blvd., Sinjhuang Dist., New Taipei City 24250, Taiwan |
| 16 | Chi Formula | Powder | No.676, Chongde Rd., Dong Dist., Tainan 70173, Taiwan | Hsinten International Group 7F., No.217, Sec.2, Sinbei Blvd., Sinjhuang Dist., New Taipei City 24250, Taiwan |
| 17 | Key Point | Tablet | No.676, Chongde Rd., Dong Dist., Tainan 70173, Taiwan | Hsinten International Group 7F., No.217, Sec.2, Sinbei Blvd., Sinjhuang Dist., New Taipei City 24250, Taiwan |
| 18 | Chinese medicine pill(black) | Chinese medicine pill (black) | No.2, Niouliao, Tianliao Dist., Kaohsiung 82343, Taiwan | No.2, Niouliao, Tianliao Dist., Kaohsiung 82343, Taiwan |
| 19 | Eye Drop(Indomen) | Eye drop | No.11, Changrong, Jiangjyun Dist., Tainan 72541, Taiwan | No.11, Changrong, Jiangjyun Dist., Tainan 72541, Taiwan |
| 20 | Solution(Indomen) | Solution | No.11, Changrong, Jiangjyun Dist., Tainan 72541, Taiwan | No.11, Changrong, Jiangjyun Dist., Tainan 72541, Taiwan |
| 21 | Dan-shen Root | Chinese medicine powder | No.315, Jialising, Jiali Dist., Tainan 72262, Taiwan | No.315, Jialising, Jiali Dist., Tainan 72262, Taiwan |
| 22 | American Ginseng | Chinese medicine powder | No.15-2, Wunzihnei, Jiali Dist., Tainan 72269, Taiwan | No.15-2, Wunzihnei, Jiali Dist., Tainan 72269, Taiwan |
| 23 | Figwort Root and Blackberrglily Rhizome | Chinese medicine powder | No.158, Yanping Rd., Jiali Dist., Tainan 72241, Taiwan | No.158, Yanping Rd., Jiali Dist., Tainan 72241, Taiwan |
| 24 | BestVite L-Arginine tablet | Tablet | momo.com Inc. | momo.com Inc. |
| 25 | Chinese medicine powder | Chinese medicine powder | No.203, Jianguo Rd., Jhong Dist., Taichung 40043, Taiwan | No.203, Jianguo Rd., Jhong Dist., Taichung 40043, Taiwan |
| 26 | Yunfeng Pavilion Tea Mind | herbal tea | No.10-5, Shueihuodong, Baihe Dist., Tainan 73255, Taiwan | No.10-5, Shueihuodong, Baihe Dist., Tainan 73255, Taiwan |
| 27 | Chinese Medicine Ointment | Ointment | No.410, Sec.2, Jiankang Rd., Nan Dist., Tainan 70262, Taiwan | No.410, Sec.2, Jiankang Rd., Nan Dist., Tainan 70262, Taiwan |
| 28 | Chinese medicine capsule | Chinese medicine capsule | No.6-16, Jiaba, Shanhua Dist., Tainan 74173, Taiwan | No.6-16, Jiaba, Shanhua Dist., Tainan 74173, Taiwan |
| 29 | Xiangruyi Burn Ointment | Ointment | No.41, Ln.260, Dingan St., Annan Dist., Tainan 70944, Taiwan | No.41, Ln.260, Dingan St., Annan Dist., Tainan 70944, Taiwan |
| 30 | Xiangruyi Burn Wound Patch | Wound Patch | No.41, Ln.260, Dingan St., Annan Dist., Tainan 70944, Taiwan | No.41, Ln.260, Dingan St., Annan Dist., Tainan 70944, Taiwan |
| 31 | EGCG Capsules | Capsule | No.777, Wunsian Rd., Bei Dist., Tainan 70459, Taiwan | No.777, Wunsian Rd., Bei Dist., Tainan 70459, Taiwan |
| 32 | Capsules | Capsule | No.350, Jhonghua 2nd Rd., Yongkang Dist., Tainan 71079, Taiwan | No.350, Jhonghua 2nd Rd., Yongkang Dist., Tainan 71079, Taiwan |
| 33 | Fairy Maiden Capsules | Capsule | No.139, Huasing St., Yongkang Dist., Tainan 71069, Taiwan | Baixing Biotechnoloby Co., Ltd. No.73, Ln.221, Yude Rd., Bei Dist., Tainan 70450, Taiwan |
| 34 | Chinese medicine powder | Chinese medicine powder | No.18, Ln.114, Dade Rd., Gueiren Dist., Tainan 71144, Taiwan | No.18, Ln.114, Dade Rd., Gueiren Dist., Tainan 71144, Taiwan |
| 35 | Chinese medicine pill | Chinese medicine pill | No.18, Ln.114, Dade Rd., Gueiren Dist., Tainan 71144, Taiwan | No.18, Ln.114, Dade Rd., Gueiren Dist., Tainan 71144, Taiwan |
| 36 | Chinese medicine powder | Chinese medicine powder | No.18, Ln.114, Dade Rd., Gueiren Dist., Tainan 71144, Taiwan | No.18, Ln.114, Dade Rd., Gueiren Dist., Tainan 71144, Taiwan |
| 37 | Chinese medicine pill | Chinese medicine pill | No.18, Ln.114, Dade Rd., Gueiren Dist., Tainan 71144, Taiwan | No.18, Ln.114, Dade Rd., Gueiren Dist., Tainan 71144, Taiwan |
| 38 | Chinese medicine powder | Chinese medicine powder | No.18, Ln.114, Dade Rd., Gueiren Dist., Tainan 71144, Taiwan | No.18, Ln.114, Dade Rd., Gueiren Dist., Tainan 71144, Taiwan |
| 39 | Chinese medicine powder | Chinese medicine powder | No.18, Ln.114, Dade Rd., Gueiren Dist., Tainan 71144, Taiwan | No.18, Ln.114, Dade Rd., Gueiren Dist., Tainan 71144, Taiwan |
| 40 | Chinese medicine powder | Chinese medicine powder | No.18, Ln.114, Dade Rd., Gueiren Dist., Tainan 71144, Taiwan | No.18, Ln.114, Dade Rd., Gueiren Dist., Tainan 71144, Taiwan |
| 41 | Chinese medicine capsule | Chinese medicine capsule | No.18, Ln.114, Dade Rd., Gueiren Dist., Tainan 71144, Taiwan | No.18, Ln.114, Dade Rd., Gueiren Dist., Tainan 71144, Taiwan |
| 42 | Chinese medicine powder | Chinese medicine powder | No.18, Ln.114, Dade Rd., Gueiren Dist., Tainan 71144, Taiwan | No.18, Ln.114, Dade Rd., Gueiren Dist., Tainan 71144, Taiwan |
| 43 | Chinese medicine powder | Chinese medicine powder | No.18, Ln.114, Dade Rd., Gueiren Dist., Tainan 71144, Taiwan | No.18, Ln.114, Dade Rd., Gueiren Dist., Tainan 71144, Taiwan |
| 44 | Chinese medicine capsule(greeen) | Chinese medicine capsule (greeen) | No.18, Ln.114, Dade Rd., Gueiren Dist., Tainan 71144, Taiwan | No.18, Ln.114, Dade Rd., Gueiren Dist., Tainan 71144, Taiwan |
| 45 | Chinese medicine capsule(red-white) | Chinese medicine capsule (red-white) | No.18, Ln.114, Dade Rd., Gueiren Dist., Tainan 71144, Taiwan | No.18, Ln.114, Dade Rd., Gueiren Dist., Tainan 71144, Taiwan |
| 46 | Chinese medicine pill | Chinese medicine pill | No.18, Ln.114, Dade Rd., Gueiren Dist., Tainan 71144, Taiwan | No.18, Ln.114, Dade Rd., Gueiren Dist., Tainan 71144, Taiwan |
| 47 | Chinese medicine pill | Chinese medicine pill | No.18, Ln.114, Dade Rd., Gueiren Dist., Tainan 71144, Taiwan | No.18, Ln.114, Dade Rd., Gueiren Dist., Tainan 71144, Taiwan |
| 48 | Chinese medicine pill | Chinese medicine pill | No.18, Ln.114, Dade Rd., Gueiren Dist., Tainan 71144, Taiwan | No.18, Ln.114, Dade Rd., Gueiren Dist., Tainan 71144, Taiwan |
| 49 | Chinese medicine pill | Chinese medicine pill | No.18, Ln.114, Dade Rd., Gueiren Dist., Tainan 71144, Taiwan | No.18, Ln.114, Dade Rd., Gueiren Dist., Tainan 71144, Taiwan |
| 50 | Chinese medicine pill | Chinese medicine pill | No.18, Ln.114, Dade Rd., Gueiren Dist., Tainan 71144, Taiwan | No.18, Ln.114, Dade Rd., Gueiren Dist., Tainan 71144, Taiwan |
| 51 | Chinese medicine pill | Chinese medicine pill | No.18, Ln.114, Dade Rd., Gueiren Dist., Tainan 71144, Taiwan | No.18, Ln.114, Dade Rd., Gueiren Dist., Tainan 71144, Taiwan |
| 52 | Chinese medicine pill | Chinese medicine pill | No.18, Ln.114, Dade Rd., Gueiren Dist., Tainan 71144, Taiwan | No.18, Ln.114, Dade Rd., Gueiren Dist., Tainan 71144, Taiwan |
| 53 | Chinese medicine pill | Chinese medicine pill | No.18, Ln.114, Dade Rd., Gueiren Dist., Tainan 71144, Taiwan | No.18, Ln.114, Dade Rd., Gueiren Dist., Tainan 71144, Taiwan |
| 54 | Chinese medicine pill | Chinese medicine pill | No.18, Ln.114, Dade Rd., Gueiren Dist., Tainan 71144, Taiwan | No.18, Ln.114, Dade Rd., Gueiren Dist., Tainan 71144, Taiwan |
| 55 | Chinese medicine pill | Chinese medicine pill | No.18, Ln.114, Dade Rd., Gueiren Dist., Tainan 71144, Taiwan | No.18, Ln.114, Dade Rd., Gueiren Dist., Tainan 71144, Taiwan |
| 56 | Chinese medicine pill | Chinese medicine pill | No.18, Ln.114, Dade Rd., Gueiren Dist., Tainan 71144, Taiwan | No.18, Ln.114, Dade Rd., Gueiren Dist., Tainan 71144, Taiwan |
| 57 | Chinese medicine pill | Chinese medicine pill | No.18, Ln.114, Dade Rd., Gueiren Dist., Tainan 71144, Taiwan | No.18, Ln.114, Dade Rd., Gueiren Dist., Tainan 71144, Taiwan |
| 58 | Chinese medicine powder | Chinese medicine powder | No.12-1, Ln.647, Siaodong Rd., Yongkang Dist., Tainan 71052, Taiwan | No.12-1, Ln.647, Siaodong Rd., Yongkang Dist., Tainan 71052, Taiwan |
| 59 | Mega high softgels | Soft capsule | No.785, Sec.2, Yonghua Rd., Anping Dist., Tainan 70848, Taiwan | PCW Nutritional Company Northridge, CA, USA |
| 60 | KGCheck Shaping great healthy capsule | Soft capsule | 1F., No.760, Sec.2, Yonghua Rd., Anping Dist., Tainan 70848, Taiwan | LianHwa Foods Corp. |
| 61 | Ah Torng Bor Slimming Film Coated Tablet | Film Coated Tablet | No.57, Sec.2, Datong Rd., Dong Dist., Tainan 70150, Taiwan | Ah Torng Bor Biotechnology pharm. CO., LTD |
| 62 | Youbest 3 in 1 soft capsules | Soft capsule | No.57, Sec.2, Datong Rd., Dong Dist., Tainan 70150, Taiwan | Sinphar Group |
| 63 | Fiber miracle | Powder | No.57, Sec.2, Datong Rd., Dong Dist., Tainan 70150, Taiwan | NuAra Biotech |
| 64 | Goodlife Maca soft capsule | Soft capsule | No.163, Sec.1, Datong Rd., Dong Dist., Tainan 70150, Taiwan | 9F.-1, No.296, Sec.5, Nanjing E. Rd., Songshan Dist., Taipei 10569, Taiwan |
| 65 | Chitosan capsule | Soft capsule | No.163, Sec.1, Datong Rd., Dong Dist., Tainan 70150, Taiwan | No.163, Sec.1, Datong Rd., Dong Dist., Tainan 70150, Taiwan |
| 66 | C.L.A soft capsule | Soft capsule | No.163, Sec.1, Datong Rd., Dong Dist., Tainan 70150, Taiwan | No.163, Sec.1, Datong Rd., Dong Dist., Tainan 70150, Taiwan |
| 67 | Sun power smooth | Tablet | 1F., No.62, Jhongjheng N. Rd., Yongkang Dist., Tainan 71072, Taiwan | 1F., No.512, Sec.2, Wucyuan W. Rd., Nantun Dist., Taichung 40878, Taiwan |
| 68 | Beauty Ahriman Tablets | Tablet | 1F., No.62, Jhongjheng N. Rd., Yongkang Dist., Tainan 71072, Taiwan | YougShin Group |
| 69 | DV-go Slender tablet | Tablet | No.133, Sec.3, Jhongshan Rd., Gueiren Dist., Tainan 71152, Taiwan | 9F.-1, No.296, Sec.5, Nanjing E. Rd., Songshan Dist., Taipei 10569, Taiwan |
| 70 | Capsules | Soft capsule | No.151, Sec.2, Jhongshan Rd., Gueiren Dist., Tainan 71146, Taiwan |  |
| 71 | Meibeaut capsules | Soft capsule | 1F., No.60, Sec.4, Jinhua Rd., Jhongsi Dist., Tainan 70059, Taiwan | 1F., No.60, Sec.4, Jinhua Rd., Jhongsi Dist., Tainan 70059, Taiwan |
| 72 | Super female cpasules | Soft capsule | 1F., No.60, Sec.4, Jinhua Rd., Jhongsi Dist., Tainan 70059, Taiwan | 1F., No.60, Sec.4, Jinhua Rd., Jhongsi Dist., Tainan 70059, Taiwan |
| 73 | Ko lin tun yung chiu capsules | Soft capsule | No.371, Guangfu Rd., Jiali Dist., Tainan 72257, Taiwan | PCW Nutritional Company Northridge, CA, USA |
| 74 | DH-Carnitine 120 T | Soft capsule | No.17-22, Jinsyue Rd., Jiali Dist., Tainan 72252, Taiwan | DHBIO Company Limited |
| 75 | Golden samurai softgels | Soft capsule | No.27, Sec.1, Sindong Rd., Dongshan Dist., Tainan 73351, Taiwan | VIVA pharmaceutical. INC, Richmond, CANADA |
| 76 | Young 99 | Tablet | No.23, Jhongsing Rd., Dongshan Dist., Tainan 73343, Taiwan | OUYeh pharmaceutical Co., Ltd |
| 77 | LaLa | Powder | No.319-1, Jhongjheng Rd., Sinshih Dist., Tainan 74447, Taiwan | Biozyme Biotechnology Co., Ltd. |
| 78 | KGCheck Shaping great healthy capsule | Soft capsule | No.318, Renai St., Sinshih Dist., Tainan 74441, Taiwan | LianHwa Foods Corp. |
| 79 | Ophiocordyceps sinensis | Chinese medicine powder | - | - |
| 80 | Antrodia camphorata | Chinese medicine powder | - | - |
