# Supplementary material for: Detection of adulterated drugs in traditional Chinese medicine and dietary supplements using hydrogen as a carrier gas
Source: PLoS One. 2018 Oct 10;13(10):e0205371. doi: 10.1371/journal.pone.0205371 (PMC6179279; doi:10.1371/journal.pone.0205371)
Supplement: S2 Table — (DOCX) [file pone.0205371.s002.docx]

**S2 Table. Information of standard**

| Standard Number | Compound Name | Molecular Weight | Purity (%) | Brand | Lot Number | Storage | Solvent | Source Origin |
| --- | --- | --- | --- | --- | --- | --- | --- | --- |
| 1 | Acetaminophen | 151.00 | 99.8% | USP | K0I244 | -20℃ | 95%Ethanol | Rockville, MD, USA |
| 2 | Acetildenafil | 466.59 | 99.1% | TLC | 1218-018A11 | -20℃ | 98%Ethanol | Aurora, Ontario, Canada |
| 3 | Acetohexamide | 324.00 | 100.0% | USP | 0600 | RT | Ethanol | Rockville, MD, USA |
| 4 | Allopurinol | 136.00 | 100.0% | USP | J0C186 | -20℃ | 95%Ethanol | Rockville, MD, USA |
| 5 | Aminopyrine | 231.29 | 100.0% | Sigma-Aldrich | 020M1618V | -20℃ | 98%Ethanol | Saint Louis, MO, USA |
| 6 | Aminotadalafil | 390.40 | 99.9% | TLC | 1188-024A1 | -20℃ | 98%Ethanol | Aurora, Ontario, Canada |
| 7 | Amitripthyline | 313.90 | 100.0% | USP | K0L010 | -20℃ | 95%Ethanol | Rockville, MD, USA |
| 8 | Amphetamine（1mg/mL） | 135.21 | 99.8% | Cerilliant | FE072712-02 | -20℃ | Methanol | Round Rock, Texas, USA |
| 9 | Aspirin | 180.00 | 100.0% | USP | H | -20℃ | 95%Ethanol | Rockville, MD, USA |
| 10 | Atenolol | 266.00 | 99.9% | USP | I0F032 | -20℃ | 95%Ethanol | Rockville, MD, USA |
| 11 | Atropine | 694.83 | 99.7% | USP | N0H159 | -20℃ | 95%Ethanol | Rockville, MD, USA |
| 12 | Barbital | 184.19 | 100.0% | Sigma-Aldrich | SLBN5978V | -20℃ | 98%Ethanol | Saint Louis, MO, USA |
| 13 | Benzbromarone | 424.00 | 100.0% | European Pharmacopoeia | 1 | -20℃ | 98%Ethanol | Strasbourg, France |
| 14 | Benzocaine | 165.00 | 100.0% | USP | J1G214 | RT | Ethanol | Rockville, MD, USA |
| 15 | Betamethasone | 392.00 | 99.3% | USP | L0G059 | -20℃ | 95%Ethanol | Rockville, MD, USA |
| 16 | Bezafibrate | 361.00 | 100.0% | European Pharmacopoeia | 1.2 | -20℃ | 98%Ethanol | Strasbourg, France |
| 17 | Bisacodyl | 361.00 | 99.7% | USP | J0G408 | -20℃ | 95%Ethanol | Rockville, MD, USA |
| 18 | Bromhexine | 376.00 | 100.0% | Euro Chem-Pharma | 00NVLO | 4℃ | Ethanol | Penang, Malaysia |
| 19 | Brompheniramine | 435.31 | 99.6% | USP | J0H148 | -20℃ | 95%Ethanol | Rockville, MD, USA |
| 20 | Bromvalerylurea | 223.07 | 98.0% | TCI | TTRSA-TK | -20℃ | 95%Ethanol | Tokyo, Japan |
| 21 | Bucetin | 223.27 | 99.0% | Sigma-Aldrich | 060h0913v | -20℃ | 98%Ethanol | Saint Louis, MO, USA |
| 22 | Caffeine | 194.19 | 100.0% | Fluka | LRAB0279 | RT | 98%Ethanol | Laramie, WY, USA |
| 23 | Carbetapentane | 525.59 | 98.0% | Sigma-Aldrich | 023H7711V | 4℃ | Ethanol | Saint Louis, MO, USA |
| 24 | Carbimazole | 186.00 | 99.7% | CRS | 1054 | -20℃ | 95%Ethanol | Canoga Park, CA, US |
| 25 | Carbinoxamine | 406.86 | 99.9% | USP | H1G030 | -20℃ | 95%Ethanol | Rockville, MD, USA |
| 26 | Carbodenafil | 452.56 | 99.0% | TLC | 1087-063A4 | -20℃ | 98%Ethanol | Aurora, Ontario, Canada |
| 27 | Carisoprodol（1mg/mL） | 260.00 | 98.6% | Cerilliant | FN110810-01 | -20℃ | Methanol | Round Rock, Texas, USA |
| 28 | Chloramphenicol | 322.00 | 99.8% | Sigma-Aldrich | SZB8294XV | RT | Ethanol | Saint Louis, MO, USA |
| 29 | Chlordiazepoxide（1mg/mL） | 299.75 | 98.5% | Cerilliant | FE062310-01 | -20℃ | Methanol | Round Rock, Texas, USA |
| 30 | Chlormezanone | 273.00 | 99.9% | TCI | GL01 | RT | Ethanol | Tokyo, Japan |
| 31 | Chlorpheniramine Maleate（1mg/mL） | 390.86 | 99.6% | Cerilliant | FN110410-04 | -20℃ | Methanol | Round Rock, Texas, USA |
| 32 | Chlorpromazine | 355.33 | 100.0% | USP | J1K257 | -20℃ | 95%Ethanol | Rockville, MD, USA |
| 33 | Chlorpropamide | 276.00 | 99.3% | USP | I0H184 | -20℃ | 95%Ethanol | Rockville, MD, USA |
| 34 | Chlorzoxazone | 169.00 | 99.8% | USP | J0H074 | -20℃ | 95%Ethanol | Rockville, MD, USA |
| 35 | Cimetidine | 252.00 | 99.7% | USP | J0H291 | -20℃ | 95%Ethanol | Rockville, MD, USA |
| 36 | Cinnarizine | 368.50 | 100.0% | USP | 00AZ25 | -20℃ | 95%Ethanol | Rockville, MD, USA |
| 37 | Clobenzorex hydrochloride | 296.20 | 99.2% | National Measurement Institute | 09-D-09 | -20℃ | 98%Ethanol | Sydney, NSW, Australia |
| 38 | Clofibrate | 242.00 | 100.0% | USP | I | -20℃ | 95%Ethanol | Rockville, MD, USA |
| 39 | Cocaine（1mg/mL） | 303.00 | 101.3% | Cerilliant | FE051012-01 | -20℃ | Acetonitrile | Round Rock, Texas, USA |
| 40 | Colchicine | 399.00 | 99.4% | USP | L0H214 | -20℃ | 95%Ethanol | Rockville, MD, USA |
| 41 | Cortisone | 402.48 | 100.0% | USP | I | -20℃ | 95%Ethanol | Rockville, MD, USA |
| 42 | 7-Keto-dehydroepiandrosterone(7-keto-DHEA) | 304.43 | 98.0% | AK Scientific | JL30533 | -20℃ | 98%Ethanol | Union City, CA, USA |
| 43 | N-Desmethylsibutramine | 402.29 | 98.3% | TLC | 1082-067A1 | -20℃ | 98%Ethanol | Aurora, Ontario, Canada |
| 44 | N-Didesmethylsibutramine | 288.26 | 98.1% | TLC | 1037-027A1 | -20℃ | 98%Ethanol | Aurora, Ontario, Canada |
| 45 | Dexamethasone | 392.00 | 99.7% | USP | K0H243 | -20℃ | 95%Ethanol | Rockville, MD, USA |
| 46 | Dextromethorphan | 370.32 | 100.0% | USP | K0F118 | -20℃ | 98%Ethanol | Rockville, MD, USA |
| 47 | Diazepam（1mg/mL） | 284.74 | 100.4% | Cerilliant | FE082310-03 | -20℃ | Methanol | Round Rock, Texas, USA |
| 48 | Dibucaine | 379.92 | 99.9% | USP | J0I022 | -20℃ | 95%Ethanol | Rockville, MD, USA |
| 49 | Diclofenac Sodium | 318.13 | 99.9% | USP | H2K051 | -20℃ | 95%Ethanol | Rockville, MD, USA |
| 50 | Dicyclomine | 345.95 | 100.0% | USP | H | -20℃ | 95%Ethanol | Rockville, MD, USA |
| 51 | Diethylpropion HCl（1mg/mL） | 205.30 | 100.1% | Cerilliant | FE012312-03 | -20℃ | 1% 1 M HCl in Methanol | Round Rock, Texas, USA |
| 52 | Diethylstilbestrol | 268.00 | 100.0% | USP | K5B291 | -20℃ | 95%Ethanol | Rockville, MD, USA |
| 53 | Dimethylsildenafil | 488.61 | 99.5% | TLC | 1215-003A2 | -20℃ | 98%Ethanol | Aurora, Ontario, Canada |
| 54 | Diphenhydramine | 291.82 | 99.8% | USP | J1G210 | -20℃ | 95%Ethanol | Rockville, MD, USA |
| 55 | Diphenylhydantoin | 252.27 | 100.0% | USP | J0E090 | -20℃ | 95%Ethanol | Rockville, MD, USA |
| 56 | Diprophylline | 254.00 | 100.0% | USP | G-2 | -20℃ | 95%Ethanol | Rockville, MD, USA |
| 57 | Econazole | 444.70 | 100.0% | USP | H0K380 | -20℃ | 95%Ethanol | Rockville, MD, USA |
| 58 | Estradiol benzoate | 376.00 | 100.0% | USP | H0C332 | -20℃ | 95%Ethanol | Rockville, MD, USA |
| 59 | Estriol | 288.00 | 100.0% | USP | L0K175 | -20℃ | 95%Ethanol | Rockville, MD, USA |
| 60 | Estrone | 270.37 | 100.0% | USP | L0F119 | -20℃ | 95%Ethanol | Rockville, MD, USA |
| 61 | Ethinylestradiol | 296.00 | 100.0% | USP | Q0C162 | -20℃ | 95%Ethanol | Rockville, MD, USA |
| 62 | Ethisterone | 312.45 | 98.5% | Sigma-Aldrich | SZB9156XV | -20℃ | 95%Ethanol | Saint Louis, MO, USA |
| 63 | Ethoxybenzamide | 165.00 | 99.8% | Sigma-Aldrich | STBC0011V | RT | Ethanol | Saint Louis, MO, USA |
| 64 | Ethylestrenol | 288.47 | 100.0% | AApin | N22991e | -20℃ | 95%Ethanol | Abingdon, Oxon, UK |
| 65 | Fenfluramine（1mg/mL） | 231.26 | 101.6% | Cerilliant | FE050211-01 | -20℃ | Methanol | Round Rock, Texas, USA |
| 66 | Finasteride | 372.54 | 100.0% | USP | G0K072 | -20℃ | 95%Ethanol | Rockville, MD, USA |
| 67 | Flavoxate | 427.92 | 100.0% | USP | F0H157 | -20℃ | 95%Ethanol | Rockville, MD, USA |
| 68 | Fluoxetine | 345.79 | 99.9% | USP | G0F085 | -20℃ | 95%Ethanol | Rockville, MD, USA |
| 69 | Fluoxymesterone（1mg/mL） | 336.44 | 100.0% | Cerilliant | FE06021502 | -20℃ | 1,2-Dimethoxyethane | Round Rock, Texas, USA |
| 70 | Gemfibrozil | 250.00 | 99.7% | USP | H1F285 | -20℃ | 95%Ethanol | Rockville, MD, USA |
| 71 | Gendenafil | 354.41 | 98.8% | TLC | 1045-066A2 | -20℃ | 98%Ethanol | Aurora, Ontario, Canada |
| 72 | Griseofulvin | 352.00 | 97.4% | Sigma-Aldrich | SZBB171XV | -20℃ | 95%Ethanol | Saint Louis, MO, USA |
| 73 | Guaifenesin | 198.22 | 99.4% | USP | J0G257 | -20℃ | 98%Ethanol | Rockville, MD, USA |
| 74 | Homatropine | 356.00 | 100.0% | USP | IOE341 | RT | Ethanol | Rockville, MD, USA |
| 75 | Homosildenafil | 488.61 | 99.9% | TLC | 1031-035A1 | -20℃ | 98%Ethanol | Aurora, Ontario, Canada |
| 76 | Hydralazine | 196.64 | 100.0% | USP | M0I216 | -20℃ | 95%Ethanol | Rockville, MD, USA |
| 77 | Hydrocortisone | 362.00 | 99.7% | USP | N0F289 | -20℃ | 95%Ethanol | Rockville, MD, USA |
| 78 | Ibuprofen | 206.00 | 99.9% | USP | K0J008 | -20℃ | 95%Ethanol | Rockville, MD, USA |
| 79 | Imidazosagatriazinone | 312.37 | 99.3% | TLC | 1032-058B1 | -20℃ | 98%Ethanol | Aurora, Ontario, Canada |
| 80 | Indomethacin | 357.00 | 100.0% | USP | J1G345 | -20℃ | 95%Ethanol | Rockville, MD, USA |
| 81 | Ketoprofen | 254.00 | 99.8% | USP | H1H247 | -20℃ | 95%Ethanol | Rockville, MD, USA |
| 82 | Lidocaine | 234.00 | 99.8% | USP | M0F220 | -20℃ | 95%Ethanol | Rockville, MD, USA |
| 83 | Lorazepam（1mg/mL） | 320.00 | 99.9% | Cerilliant | FE032511-01 | -20℃ | Acetonitrile | Round Rock, Texas, USA |
| 84 | Mazindol | 284.74 | 99.9% | USP | I0H012 | -20℃ | 98%Ethanol | Rockville, MD, USA |
| 85 | Mefenamic acid | 241.00 | 100.0% | USP | G0C025 | -20℃ | 95%Ethanol | Rockville, MD, USA |
| 86 | Melatonin | 232.00 | 99.9% | USP | F0E027 | -20℃ | 95%Ethanol | Rockville, MD, USA |
| 87 | Mephenesin | 182.22 | 99.9% | Alfa Aesar | G7437A | -20℃ | 95%Ethanol | Heysham, Lancashire, UK |
| 88 | Mephentermine | 199.72 | 98.0% | TRC | 14-PSB-86-1 | -20℃ | 98%Ethanol | North York, ON, Canada |
| 89 | Meprobamate（1mg/mL） | 218.25 | 101.4% | Cerilliant | FE060412-12 | -20℃ | Methanol | Round Rock, Texas, USA |
| 90 | Methamphetamine（1mg/mL） | 149.23 | 102.6% | Cerilliant | FE082712-03 | -20℃ | Methanol | Round Rock, Texas, USA |
| 91 | Methandriol | 304.47 | 98.0% | Steraloids,lnc | F098 | -20℃ | 95%Ethanol | Newport, RI, USA |
| 92 | Methandrostenolone | 300.00 | 99.1% | Sigma-Aldrich | BCBJ8206V | 4℃ | Ethanol | Saint Louis, MO, USA |
| 93 | Methaqualone（1mg/mL） | 250.30 | 100.0% | Cerilliant | FE091410-03 | -20℃ | Methanol | Round Rock, Texas, USA |
| 94 | Metharbital | 198.00 | 100.0% | USP | 00216-1308-05 | RT | Ethanol | Rockville, MD, USA |
| 95 | Methimazole | 114.00 | 99.9% | Fluka | SZB052XV | -20℃ | 95%Ethanol | Laramie, WY, USA |
| 96 | Methylprednisolone | 374.00 | 99.5% | USP | I0E170 | -20℃ | 95%Ethanol | Rockville, MD, USA |
| 97 | Methyltestosterone | 302.45 | 99.5% | Sigma-Aldrich | SZBC107XV | -20℃ | 98%Ethanol | Saint Louis, MO, USA |
| 98 | Metoclopramide | 354.27 | 99.9% | USP | H0D121 | -20℃ | 95%Ethanol | Rockville, MD, USA |
| 99 | Metronidazole | 171.00 | 100.0% | USP | J1I272 | -20℃ | 95%Ethanol | Rockville, MD, USA |
| 100 | Minoxidil | 209.00 | 99.9% | USP | I0K038 | -20℃ | 95%Ethanol | Rockville, MD, USA |
| 101 | Morphine（1mg/mL） | 285.34 | 97.8% | Cerilliant | FE080411-01 | -20℃ | Methanol | Round Rock, Texas, USA |
| 102 | Nalidixic acid | 232.24 | 100.0% | USP | H0I155 | -20℃ | 95%Ethanol | Rockville, MD, USA |
| 103 | Nandrolone（1mg/mL） | 274.40 | 101.6% | Cerilliant | FE051611-01 | -20℃ | Acetonitrile | Round Rock, Texas, USA |
| 104 | Naproxen | 230.00 | 99.9% | USP | J0H044 | -20℃ | 95%Ethanol | Rockville, MD, USA |
| 105 | Nifedipine | 346.00 | 99.7% | USP | L0J059 | -20℃ | 95%Ethanol | Rockville, MD, USA |
| 106 | Noracetildenafil | 452.56 | 99.9% | TLC | 1216-005A4 | -20℃ | 98%Ethanol | Aurora, Ontario, Canada |
| 107 | Norethisterone | 298.42 | 100.0% | USP | L0F237 | -20℃ | 95%Ethanol | Rockville, MD, USA |
| 108 | Orphenadrine | 461.50 | 100.0% | USP | G | -20℃ | 95%Ethanol | Rockville, MD, USA |
| 109 | Oxethazaine | 467.00 | 99.0% | Sigma-Aldrich | 048F0668V | RT | Ethanol | Saint Louis, MO, USA |
| 110 | Oxymetholone | 332.48 | 100.0% | European Pharmacopoeia | Y0001420-1.0 | 4℃ | Ethanol | Strasbourg, France |
| 111 | Oxyphenbutazone | 324.00 | 98.0% | TRC | 8-KPA-122-1 | ﹣20℃ | Ethanol | North York, ON, Canada |
| 112 | Pentazocine（1mg/mL） | 285.42 | 99.6% | Cerilliant | FE120110-01 | -20℃ | Methanol | Round Rock, Texas, USA |
| 113 | Phenacetin | 179.00 | 100.0% | USP | H-1 | -20℃ | 95%Ethanol | Rockville, MD, USA |
| 114 | Phenazopyridine | 249.70 | 99.8% | USP | H0C426 | -20℃ | 95%Ethanol | Rockville, MD, USA |
| 115 | Phenformin | 241.72 | 100.0% | USP | 0373-G | -20℃ | 95%Ethanol | Rockville, MD, USA |
| 116 | Phenobarbital（1mg/mL） | 232.24 | 99.8% | Cerilliant | FE080111-01 | -20℃ | Methanol | Round Rock, Texas, USA |
| 117 | Phenolphthalein | 318.00 | 100.0% | USP | 52490 | RT | Ethanol | Rockville, MD, USA |
| 118 | Phentermine（1mg/mL） | 149.24 | 99.9% | Cerilliant | FE012411-01 | -20℃ | Methanol | Round Rock, Texas, USA |
| 119 | Phentolamine Hydrochloride | 317.50 | 100.0% | USP | F | -20℃ | 98%Ethanol | Rockville, MD, USA |
| 120 | Phenylbutazone | 308.00 | 99.3% | USP | K0G199 | -20℃ | 95%Ethanol | Rockville, MD, USA |
| 121 | Phenylephrine | 203.67 | 99.9% | USP | L0F273 | -20℃ | 95%Ethanol | Rockville, MD, USA |
| 122 | Phenylpropanolamine HCl（1mg/mL） | 187.67 | 100.4% | Cerilliant | FN113012-04 | -20℃ | Methanol | Round Rock, Texas, USA |
| 123 | Piperidenafil | 459.57 | 99.5% | TLC | 1217-011A2 | -20℃ | 98%Ethanol | Aurora, Ontario, Canada |
| 124 | Pirenzepine | 424.32 | 99.0% | Sigma-Aldrich | 079K1733V | RT | Ethanol | Saint Louis, MO, USA |
| 125 | Piroxicam | 331.00 | 99.8% | USP | I0K351 | -20℃ | 95%Ethanol | Rockville, MD, USA |
| 126 | Prednisolone | 360.00 | 100.0% | USP | N1J277 | -20℃ | 95%Ethanol | Rockville, MD, USA |
| 127 | Prednisone | 358.00 | 99.5% | USP | O0G356 | -20℃ | 95%Ethanol | Rockville, MD, USA |
| 128 | Primidone | 218.00 | 100.0% | Sigma-Aldrich |  | RT | Ethanol | Saint Louis, MO, USA |
| 129 | Probenecid | 285.36 | 100.0% | USP | I0A011 | -20℃ | 95%Ethanol | Rockville, MD, USA |
| 130 | Procaine | 272.77 | 99.9% | Sigma-Aldrich | SZBB283XV | RT | Ethanol | Saint Louis, MO, USA |
| 131 | Progesterone | 314.00 | 99.8% | USP | IJ129 | -20℃ | 95%Ethanol | Rockville, MD, USA |
| 132 | Propantheline | 448.39 | 99.3% | USP | J0G354 | -20℃ | 95%Ethanol | Rockville, MD, USA |
| 133 | Propranolol | 295.80 | 99.9% | USP | I1G348 | -20℃ | 95%Ethanol | Rockville, MD, USA |
| 134 | Quinine | 324.00 | 97.8% | Fluka | BCBG6955V | RT | Ethanol | Laramie, WY, USA |
| 135 | Ranitidine | 350.86 | 100.0% | USP | H1G103 | RT | Ethanol | Rockville, MD, USA |
| 136 | Rimonabant Hydrochloride | 500.25 | 98.0% | TRC | 9-YM-46-1 | -20℃ | 98%Ethanol | North York, ON, Canada |
| 137 | Salicylamide | 137.00 | 100.0% | USP | F-4 | -20℃ | 95%Ethanol | Rockville, MD, USA |
| 138 | Salicylic acid | 138.00 | 99.8% | USP | K0F112 | -20℃ | 95%Ethanol | Rockville, MD, USA |
| 139 | Scopolamine | 438.31 | 100.0% | USP | K0G033 | RT | Ethanol | Rockville, MD, USA |
| 140 | Secobarbital（1mg/mL） | 238.28 | 100.8% | Cerilliant | FE080212-02 | -20℃ | Methanol | Round Rock, Texas, USA |
| 141 | Sibutramine | 279.86 | 99.9% | TLC | 1110-014A1 | -20 ℃ | 95%Ethanol | Aurora, Ontario, Canada |
| 142 | Sildenafil | 474.58 | 98.0% | TRC | 15-ABY-7-1-PFZ | -20℃ | 98%Ethanol | North York, ON, Canada |
| 143 | Stanozolol（1mg/mL） | 328.50 | 99.9% | Cerilliant | FE010912-02 | -20℃ | 1,2-Dimethoxyethane | Round Rock, Texas, USA |
| 144 | Strychnine | 334.00 | 99.9% | Sigma-Aldrich | SZB8123XV | RT | Ethanol | Saint Louis, MO, USA |
| 145 | Sulfadiazine | 250.00 | 99.9% | USP | J1G290 | -20℃ | 95%Ethanol | Rockville, MD, USA |
| 146 | Sulfadimethoxine | 250.00 | 99.9% | USP | J1G290 | -20℃ | 95%Ethanol | Rockville, MD, USA |
| 147 | Sulfamerazine | 310.00 | 99.9% | USP | H0K048 | -20℃ | 95%Ethanol | Rockville, MD, USA |
| 148 | Sulfamethazine | 264.00 | 99.9% | USP | I0I081 | -20℃ | 95%Ethanol | Rockville, MD, USA |
| 149 | Sulfamethoxazole | 253.00 | 99.8% | USP | J0F148 | -20℃ | 95%Ethanol | Rockville, MD, USA |
| 150 | Sulfamethoxypyridazine | 280.00 | 99.6% | Sigma-Aldrich | 050H0647V | 4℃ | Ethanol | Saint Louis, MO, USA |
| 151 | Sulfanilamide | 172.00 | 100.0% | USP | O1H320 | -20℃ | 95%Ethanol | Rockville, MD, USA |
| 152 | Sulfinpyrazone | 404.00 | 99.2% | USP | H0C416 | -20℃ | 95%Ethanol | Rockville, MD, USA |
| 153 | Sulindac | 356.00 | 100.0% | USP | IOJ345 | RT | Ethanol | Rockville, MD, USA |
| 154 | Synephrine | 167.21 | 98.8% | ChromaDex | 00019445-55D | -20 ℃ | 95%Ethanol | Irvine, CA, USA |
| 155 | Tadalafil | 389.40 | 99.9% | USP | F0L003 | -20℃ | 98%Ethanol | Rockville, MD, USA |
| 156 | Terbinafine | 327.90 | 100.0% | USP | G0K287 | -20℃ | 95%Ethanol | Rockville, MD, USA |
| 157 | Testosterone | 288.00 | 100.0% | European Pharmacopoeia | 2.1 | -20℃ | 98%Ethanol | Strasbourg, France |
| 158 | Tetracaine | 300.82 | 100.0% | USP | KOF307 | RT | Ethanol | Rockville, MD, USA |
| 159 | Theobromine | 180.17 | 100.0% | ChromaDex | A1039B | -20℃ | 95%Ethanol | Irvine, CA, USA |
| 160 | Theophylline | 180.00 | 100.0% | USP | J1H052 | -20℃ | 95%Ethanol | Rockville, MD, USA |
| 161 | Thiodimethylsildenafil | 504.68 | 99.7% | TLC | 1214-006A3 | -20℃ | 98%Ethanol | Aurora, Ontario, Canada |
| 162 | Thiohomosildenafil | 504.68 | 99.4% | TLC | 1220-033A10 | -20℃ | 98%Ethanol | Aurora, Ontario, Canada |
| 163 | Thioridazine | 407.04 | 100.0% | USP | H | -20℃ | 95%Ethanol | Rockville, MD, USA |
| 164 | Thiosildenafil | 490.65 | 99.9% | TLC | 1219-038A5 | -20℃ | 98%Ethanol | Aurora, Ontario, Canada |
| 165 | Tinidazole | 247.27 | 99.7% | Sigma-Aldrich | SZBB307XV | -20℃ | 98%Ethanol | Saint Louis, MO, USA |
| 166 | Tolbutamide | 270.00 | 100.0% | USP | I | -20℃ | 95%Ethanol | Rockville, MD, USA |
| 167 | Vardenafil analogue【2-(2-ethoxy-phenyl)-5-methyl-7-propyl-3H-imidazo(5,1-f)- (1,2,4) triazin-4-one】 | 312.37 | 98.0% | TRC | 16GHZ-176-1 | -20℃ | 98%Ethanol | North York, ON, Canada |
| 168 | Yohimbine Hydrochloride | 390.91 | 98.9% | USP | G0K385 | -20℃ | 98%Ethanol | Rockville, MD, USA |
| 169 | Zolpidem（1mg/mL） | 307.39 | 100.5% | Cerilliant | FE071811-02 | -20℃ | Methanol | Round Rock, Texas, USA |
| 170 | Cetilistat | 401.58 | 98.0% | TRC | 12-GHZ-83-1 | 4℃ | 95%Ethanol | North York, ON, Canada |
